# Associations of Exposure to Parabens During Pregnancy with Behavior in Early Childhood

**DOI:** 10.3390/toxics14030211

**Published:** 2026-02-28

**Authors:** Megan L. Woodbury, Nicholas G. Cragoe, Susan L. Schantz

**Affiliations:** Beckman Institute for Advanced Science and Technology, University of Illinois at Urbana-Champaign, Urbana, IL 61801, USA; sieg1@illinois.edu (M.L.W.); schantz@illinois.edu (S.L.S.)

**Keywords:** parabens, neurodevelopment, child behavior checklist, gestational exposure, early childhood

## Abstract

(1) Background: Few studies have examined gestational paraben exposure and early childhood neurodevelopment. We evaluated associations between gestational exposure to methyl, ethyl and propyl paraben and neurodevelopment via the Child Behavior Checklist (CBCL) administered at ages 2, 3, and 4 years. (2) Methods: Gestational exposures were assessed using pooled prenatal urine samples from five time points across pregnancy. CBCL outcomes included internalizing, externalizing, and sub-scale scores. Covariate-adjusted generalized linear regression was employed to assess individual paraben exposures. Mixture analysis was performed using Bayesian Kernel Machine Regression and Quantile g-computation. (3) Results: In individual paraben analyses, each paraben was associated with increased externalizing behaviors, particularly ethylparaben (age 2: β = 0.40, 95% CI = −0.02, 0.83; age 3: β = 0.42, 95% CI = −0.19, 0.01; age 4: β = 0.18, 95% CI = −0.34, 0.70), ADHD problems at age 2 (β = 0.21, 95% CI = 0.05, 0.37), and both aggressive behavior (β = 0.38, 95% CI = 0.01, 0.74) and oppositional defiant problems (β = 0.25, 95% CI = 0.09, 0.41) at age 3. All three parabens were also associated with a reduction in withdrawn symptoms for males, especially at age 2 (ethylparaben: β = −0.09, 95% CI = −0.01, 0.85; methylparaben: β = −0.20, 95% CI = −0.34, −0.05; propylparaben: β = −0.13, 95% CI = −0.24, −0.03). The parabens mixture was associated with elevated scores in multiple CBCL subscales, though only association with oppositional defiant scores at age 3 reached significance in both BKMR (change in score when all components are at 50th percentile values compared with their 75th percentile values = 0.15; 95% CI = 0.01, 0.29) and quantile g-computation (β = 0.33, 95% CI = 0.02, 0.65), driven primarily by ethylparaben. (4) Conclusions: Individual parabens and the paraben mixture showed significant association with domains of childhood neurodevelopment, with possible detriments especially evident (a) at earlier time points, (b) in male children, and (c) in terms of externalizing behaviors.

## 1. Introduction

Parabens are a family of aliphatic esters of p-hydroxybenzoic acid often used as preservatives in consumer products, including cosmetics, such as foundations, mascara, and nail polish, in personal care products, such as shampoos and lotions, in pharmaceuticals, and in some foods in which they naturally occur [1,2,3]. Their chemical stability and effectiveness across a wide pH range, along with their lack of odor and flavor, have seen them favored for their antimicrobial properties in a diverse array of products, often in combination with other antimicrobial agents [2,3]. Their widespread use has also led to concern about exposure via contamination of water sources [4]. A few studies in animal models have linked prenatal exposure to developmental outcomes [4,5,6,7,8], but few studies have examined this relationship in humans thus far [9]. While long thought to be safe, parabens belong to the family of chemicals known as endocrine disruptors (EDCs) [3,10,11,12], due to their disruption of signaling pathways for hormones including estrogen, testosterone, and thyroid hormones [2,3,10,13]. Other endocrine disruptors, including but not limited to multiple phenols, phthalates, organophosphates, and per- and polyfluoroalkyl substances (PFAs), have been identified as potentially hazardous to neurodevelopment through their association with poorer attention, memory, and motor development, as well as increases in anxiety and stress response [14,15,16]. Given the documented associations with neurodevelopmental outcomes of other EDCs and those seen between parabens and neurodevelopment in animal studies, it is important to follow up by examining the impact of parabens on human neurodevelopment as well.

Prenatal exposure to parabens in particular has been found to be associated with neurocognitive outcomes, including cognitive impairment [17], autism spectrum disorder [18], and attention deficit hyperactivity disorder (ADHD) [13]. Rolland et al. (2025) [19] recently found individual parabens to be associated with changes in early childhood looking behaviors, indicative of altered visual memory and reaction time. Despite some existing evidence of paraben neurotoxicity, the impact of these parabens on early childhood neurodevelopment remains less than definitive, representing a critical gap in the literature given the importance of early neurodevelopment for neurodevelopmental trajectories later in childhood [16] and adulthood [20]. In the few existing studies that address the potential impacts of paraben exposure on this crucial developmental period, higher gestational exposure to parabens has been found to be associated with poorer attention and memory [19,21], and with a higher prevalence of autism symptoms in early childhood [18].

The Illinois Kids Development Study (IKIDS) recruited pregnant people during the first trimester of pregnancy and used the Child Behavior Checklist (CBCL) to evaluate associations between prenatal exposure to ethyl-, methyl-, and propylparaben and child behavior at approximately 2, 3, and 4 years of age. The CBCL was developed for use in the United States in 1978, and has since become a well-established and widely used tool for assessing behavioral issues throughout early and middle childhood and adolescence [22,23], having been further revised in 2001 [24]. We hypothesize that elevated levels of exposure to these three paraben compounds during gestation will be associated with more behavioral problems as measured by the CBCL.

## 2. Materials and Methods

### 2.1. Study Cohort

IKIDS participants were recruited between December 2013 and March 2020 at two local obstetric clinics and gave birth at two local hospitals in Urbana, Illinois. Individuals were eligible to participate if they were at less than 15 weeks of gestation; between 18 and 40 years of age; not carrying multiples; fluent in English; willing to provide a fasting blood sample and five urine samples throughout pregnancy; did not have a child already participating in IKIDS; resided within a 30 min drive of the University of Illinois campus; their doctor had not told them they had a high-risk pregnancy; and they planned to remain in the area until the child’s first birthday. Those who decided to participate were enrolled when they were between 8 and 14 weeks of gestation. Demographics, pregnancy and health history, pregnancy symptoms, medication use, maternal verbal IQ, stress and depression, and lifestyle factors, such as smoking and alcohol use, were collected via interview shortly after enrollment and updated throughout the pregnancy. Further details on the IKIDS cohort can be found in Eick et al., 2021 [25].

**Figure 1:**
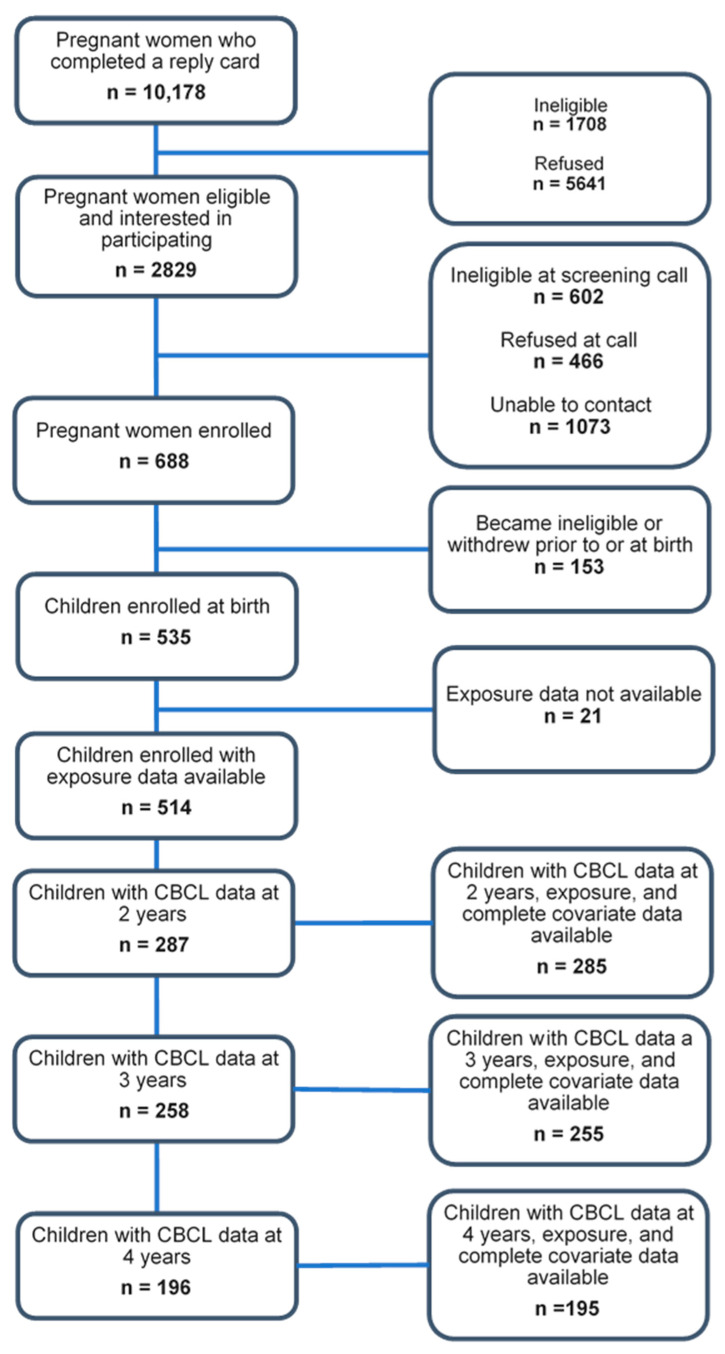
Flowchart of recruitment and retention to IKIDS study visits at 2, 3, and 4 years. Adapted from Woodbury et al. (2024) Figure 1 [33].

Cohort recruitment and retention are outlined in Figure 1. Briefly, of the 688 pregnant women enrolled in IKIDS as of March 2020, 153 withdrew or became ineligible prior to or at the time of birth, resulting in 535 infants born and enrolled in IKIDS. Of those 535 children born into the cohort, 21 did not have gestational paraben exposure data available, leaving a total of 514 children enrolled in IKIDS with gestational paraben exposure available. Demographic information for the 514 mothers with an infant enrolled in IKIDS and gestational paraben data available was generally similar to the demographic information of those who completed the CBCL at 2 (n = 285), 3 (n = 255) or 4 years of age (n = 195).

### 2.2. Quantification of Parabens by HPLC

Data for this study were abstracted from the IKIDS databank, including urinary concentrations of paraben exposure biomarkers. Participants in the IKIDS cohort were asked to provide first morning urine samples at approximately 10–14, 16–18, 22–24, 28–30, and 34–36 weeks of gestation via polypropylene collection cups. Samples were aliquoted within 48 h of collection. Specific gravity was taken for each fresh sample, after which a pooled sample consisting of 0.9 mL of urine from each sample was created for each participant, with new aliquots being added to the frozen pooled sample at each time point. At the end of pregnancy, the pooled sample was thawed and mixed, the specific gravity (SG) of the pooled sample was measured using a handheld refractometer [26,27,28,29,30], and the samples were stored at −80 °C until analysis. Median SG for the IKIDS cohort, measured using the formula Pc = P[(SG − 1)/(SGi − 1), where Pc is the measured metabolite concentration (ng/mL) and SGi is the individual sample specific gravity, was 1.016. The pooled samples were shipped overnight on dry ice to the Centers for Disease Control and Prevention (CDC) Division of Laboratory Sciences in Atlanta, GA, for quantification of paraben biomarkers using online solid-phase extraction-high-performance liquid chromatography-isotope dilution tandem mass spectrometry [31].

### 2.3. Child Behavior Measures

The CBCL is a standardized survey consisting of two composite scales (internalizing behaviors and externalizing behaviors), both of which are further divided into syndrome subscales. In addition to the internalizing and externalizing subscales, five further scales are calculated based on diagnostic criteria in the Diagnostic and Statistical Manual of Mental Disorders, Fifth Edition (DSM-5), pertaining to oppositional defiant behavior, attention deficit–hyperactivity disorder behaviors, pervasive developmental problems, anxiety problems, and affective problems. Higher scores indicate more problems in the area of behavior addressed by each question, subscale, and composite scale [32]. The internalizing behavior composite scale is composed of 36 questions and can be further subdivided into emotionally reactive (9 questions), anxious/depressed (8 questions), somatic complaints (11 questions), and withdrawn (8 questions). The externalizing behaviors composite score is composed of the sum of the scores on the attention problems (5 questions) and aggressive behavior (19 questions) subscales. Along with 7 questions pertaining to sleep problems and the remaining 33 questions categorized as other problems, the internalizing and externalizing behavior scores can be summed to calculate the total problems score [24]. The CBCL questions can also be grouped to calculate five behavioral indices, which are based on diagnostic criteria in the DSM-5. These scores cannot be considered independent of the other subscale scores because they are derived from the same questions. For example, the oppositional defiant problems scale consists of 13 questions, which are all part of the Aggressive Behavior subscale. The total problems, internalizing behavior, and externalizing behavior scores can be converted to standardized T-scores where scores above 70 are considered potentially clinically relevant [24]. For the present analysis, raw scores were used based on the recommendation in the CBCL manual that raw unstandardized scores are more appropriate for research purposes [24].

CBCL scores were derived from maternal reports. IKIDS participants were mailed surveys at 26–28, 36–38, and 48–50 months of age, and mothers mailed back the completed forms to the researchers at the University of Illinois Urbana-Champaign. For each year, a subset of participants turned in a CBCL survey; thus, the three time points contained overlapping but distinct subsamples.

### 2.4. Covariates

The following sociodemographic factors were considered for inclusion as covariates based on a priori knowledge and using directed acyclic graphing: maternal age, parity, education, verbal IQ, tobacco smoking and alcohol use during pregnancy, annual household income, child gestational age at birth, birth weight, delivery type, and age at the time of the assessment in months (see Figure S2). Maternal perceived stress score (PSS) [34] and Edinburgh Postnatal Depression Scale (EPDS) [35] scores averaged across pregnancy, infancy, and early childhood were also considered, as well as individual PSS and EPDS scores at the time of each assessment. Correlations of covariates under consideration with both exposure and outcome variables were explored, and variables were selected for inclusion when they were correlated with at least one gestational paraben exposure and at least one CBCL outcome. Included covariates closed all significant backdoor paths in the directed acyclic graph. Maternal verbal IQ and birth weight were ultimately not included as potential covariates due to the high amount of missingness. Child sex was included as a potential modifier in all models. Based on these criteria, the following covariates were included in all models: gestational age at birth, maternal age, maternal parity (nulliparous vs. ≥1), maternal education (<bachelor’s degree vs. ≥bachelor’s degree), annual household income (<$50,000, $50,000–99,999, ≥$100,000), and mean PSS and EPDS scores during pregnancy.

### 2.5. Statistical Approach

Multivariate generalized linear regression models were used to evaluate the relationship between gestational exposure to each individual paraben and raw scores for each continuous outcome at each age. Sensitivity analyses were used to evaluate the impact of additional potential confounders on the associations, including delivery type and cigarette and alcohol use during pregnancy (any vs. none). Models that excluded potential leverage points (Cook’s D > 0.06) were also examined, as well as sensitivity analyses including only children who had data available at all time points (n = 149). Descriptive and generalized linear modeling analyses were conducted using SAS Version 9.4 for Windows.

Bayesian Kernel Machine Regression (BKMR) [36,37] and quantile g-computation [38] were used to assess the potential effect of the combined exposure of all three parabens during pregnancy on CBCL scores at each age for the internalizing, externalizing, and total scores and any subscales that were associated with at least one paraben in the regression analyses. BKMR uses kernel machine regression to estimate a nonparametric, high-dimensional exposure–response function. For BKMR, continuous variables were centered and standardized prior to analysis. It was implemented with component-wide variable selection (100,000 iterations) to account for complex mixtures and identify potential interactions, and standard Markov chain Monte Carlo diagnostics were assessed for convergence [33]. Posterior inclusion probabilities (PIPs) indicate the posterior probability that an exposure is included in a model and were used to assess the importance of each paraben included in models. Those with a value >0.50 are considered important in the relationship of the mixture with the outcome. Univariate exposure–response functions were used to assess the linearity of individual parabens, while others were held constant at the 50th percentile, and interactions between the parabens were examined via bivariate exposure–response functions. The cumulative effect of exposure to the paraben mixture during pregnancy was evaluated by comparing the outcome estimates across every 5th quantile of the exposure mixture between the 25th and 75th quantiles.

Quantile g-computation uses a parametric, generalized linear model–based implementation of g-computation to estimate the effect of increasing all exposures in a mixture by one quartile on an outcome. Each exposure is assigned a positive or negative weight, depending on the direction of its association with the outcome. These weights for each outcome sum to zero, representing the proportion of the total effect contributed by the individual partial effects (positive or negative) of each exposure. Mixture analyses were conducted using R Studio version 4.3.2 using the packages bkmr (version 0.2.0) and qgcomp (version 2.8.0).

## 3. Results

### 3.1. Participation and Demographics

While more parents were white, non-Hispanic, married or living as married, had obtained at least a bachelor’s degree, had an annual household income of at least $50,000, and did not use cigarettes during pregnancy for each subsample compared to the 514 participants with exposure data available, the subsamples did not significantly differ from each other or from the full cohort (Table 1, Table 2 and Table 3; for full details, see Tables S2–S4). Each subsample also had higher maternal verbal IQ scores and lower mean PSS and EPDS scores during pregnancy and infancy, but these were also not significantly different from the full sample. Interestingly, as previously noted in this cohort [39], there was an increasing trend in PSS and EPDS scores in the subsample with both exposure and outcome data available at each age at which CBCL data were collected. Demographic information for children enrolled in the study is shown in Table 3 and Table 4, and the subsets with data at each age were similar to each other and the 514 with exposure data available, although children in each subsample tended to be slightly heavier and older at birth.

### 3.2. Gestational Paraben Exposure

Relative to participants in NHANES, paraben exposure among pregnant IKIDS participants was slightly lower, but the difference was not statistically significant (See Table 4). Methylparaben and propylparaben concentrations were above the limit of detection for 100% of participants, and ethylparaben was only below the limit of detection for 1.4% (N = 7) of participants.

### 3.3. CBCL Scores at 2, 3, and 4 Years of Age

As of April 2023, 305 children had CBCL data available at age 2, 262 at age 3, and 196 at age 4; however, not all parents provided answers to all questions; thus, not all children with CBCL data had scores for the outcomes examined in this study (Table S1). At the time of the 2-year assessment, children with data ranged in age from 25.67 to 30.90 months with an average age of 27.94 (±0.06) months (Table 5), and there were no significant differences in scores on any outcomes between males and females (Table S1).

Children with data available at the 3-year assessment were between 36.03 and 41.27 months, on average, 37.52 (±0.06) months of age, and there were some differences in scores between male and female children. Male children had higher attention problems (*p* = 0.02), ADHD Problems (*p* = 0.01), externalizing behavior (*p* = 0.03), and total problems scores (*p* = 0.03) compared to female children (Table S1). At the 4-year time point, children were between 46.37 and 58.10 months of age, with an average age of 48.09 (±0.08) months. Male children again had higher attention problems (*p* = 0.003), ADHD Problems (*p* = 0.002), and externalizing behavior scores (*p* = 0.003), but not total problems scores (*p* = 0.18), compared to female children (Table S1). Within each age range, the attention problems, ADHD problems, externalizing behavior, and total problems scores were strongly correlated (0.60 ≤ *ρ* ≤ 1.00) with one another, and the internalizing behavior score was strongly correlated with only the total problems score. Scores for each outcome across age ranges tended to be moderately to strongly correlated (0.40 ≤ *ρ* ≤ 1.00) with one another [39].

### 3.4. Associations of Gestational Paraben Exposure with CBCL Outcomes

#### 3.4.1. Ethylparaben

**Figure 2:**
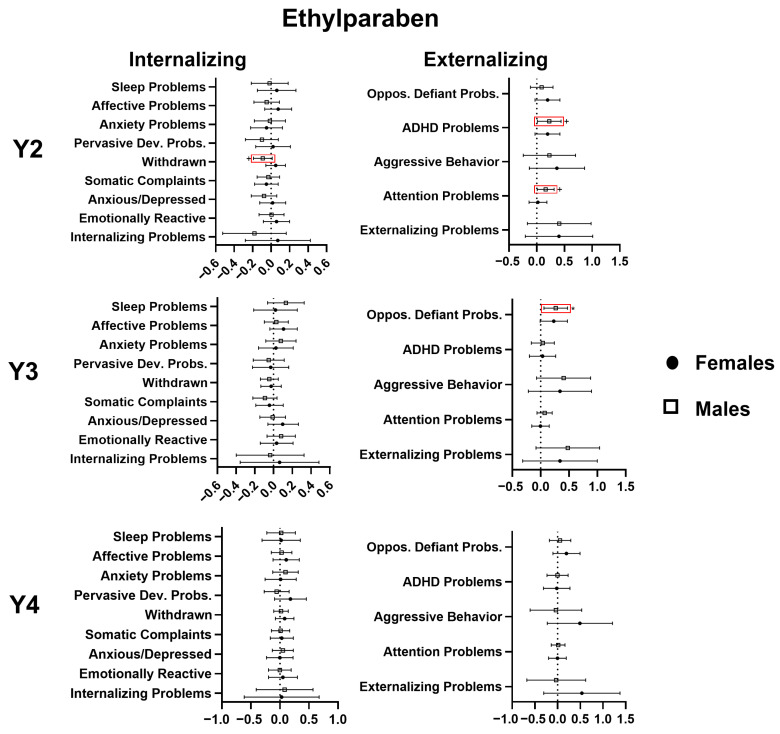
Multivariable linear regression analyses of the relation (β estimate and 95% confidence interval) of gestational ethylparaben exposure with CBCL scores at 2, 3, and 4 years of age, stratified by sex. Significant associations are marked in red. + *p* < 0.1, * *p* < 0.05.

In adjusted regression models, ethylparaben was found to be positively associated with the externalizing problems composite scale score (β = 0.405, 95% CI −0.020; 0.829, *p* < 0.1), the attention problems subscale score (β = 0.095, 95% CI 0.018; 0.208, *p* < 0.1), with the ADHD problems subscale score (β = 0.211, 95% CI 0.054; 0.369, *p* < 0.01) at age 2, and with the aggressive behavior (β = 0.377, 95% CI 0.013; 0.742, *p* < 0.05) and oppositional defiant problems subscale scores (β = 0.251; 95% CI 0.093, 0.409, *p* < 0.01) at age 3. Additionally, sex was found to be a modifier in the model pertaining to withdrawn behaviors, and in sex-stratified analysis, ethylparaben was found to be a predictor of fewer withdrawn behaviors in males at 2 years (β = −0.091, 95% CI −0.192; 0.011, *p* < 0.1). No associations were identified between ethylparaben and CBCL outcomes at age 4. For details, see Figure 2.

#### 3.4.2. Methylparaben

**Figure 3:**
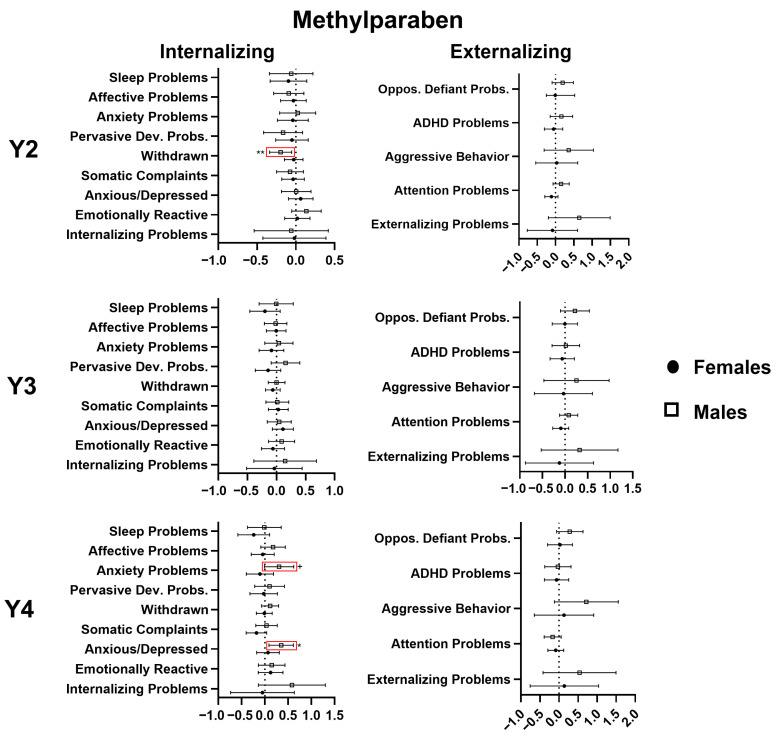
Multivariable linear regression analyses of the relation (β estimate and 95% confidence interval) of gestational methylparaben exposure with CBCL scores at 2, 3, and 4 years of age, stratified by sex. Significant associations are marked in red. + *p* < 0.1, * *p* < 0.05, ** *p* < 0.01.

At age 2, methylparaben exposure was associated with lower withdrawn behaviors subscale scores, with evidence of modification by child sex. Sex-stratified analysis revealed that the association was present only in males (−0.197, 95% CI −0.339; −0.055, *p* < 0.01). At age 3, no significant associations were identified, but at age 4, methylparaben was associated with significantly higher scores on the anxious/depressed behaviors subscale (0.353 95% CI 0.090; 0.615, *p* < 0.01) and higher anxiety subscale scores (0.306 95% CI −0.008; 0.621, *p* < 0.1) in males. For details, see Figure 3.

#### 3.4.3. Propylparaben

**Figure 4:**
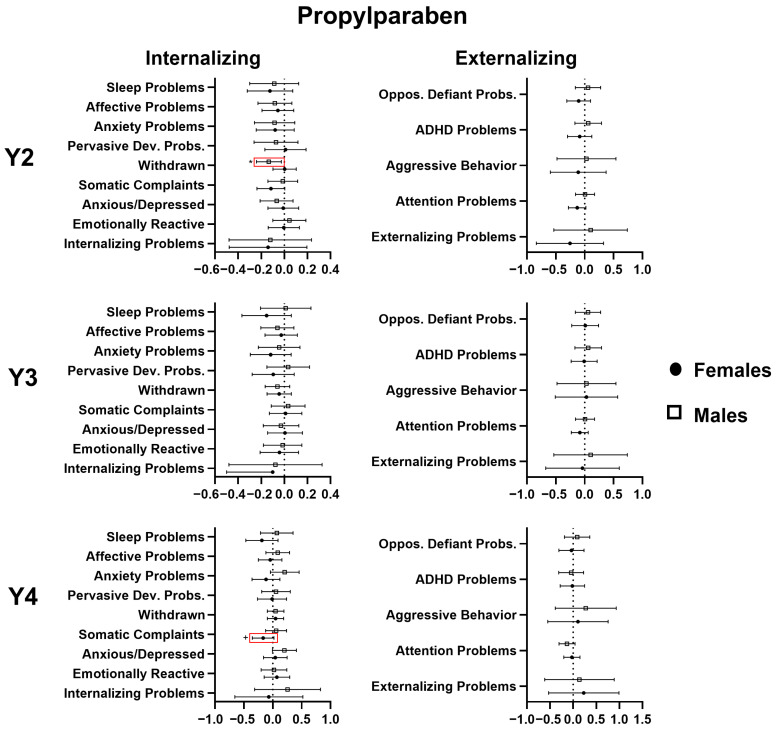
Multivariable linear regression analyses of the relation (β estimate and 95% confidence interval) of gestational propylparaben exposure with CBCL scores at 2, 3, and 4 years of age, stratified by sex. Significant associations are marked in red. + *p* < 0.1, * *p* < 0.05.

Propylparaben demonstrated relatively few associations with CBCL outcomes compared with the other parabens. At age 2, propylparaben (such as ethyl- and methylparaben) was found to be associated with decreased withdrawn behaviors in males (−0.134, 95% CI −0.241; −0.026, *p* < 0.01), and at age 4, it was associated with lower somatic complaints for females (−0.167 95% CI −0.354; 0.020, *p* < 0.1). For details, see Figure 4.

#### 3.4.4. Mixture Models

**Figure 5:**
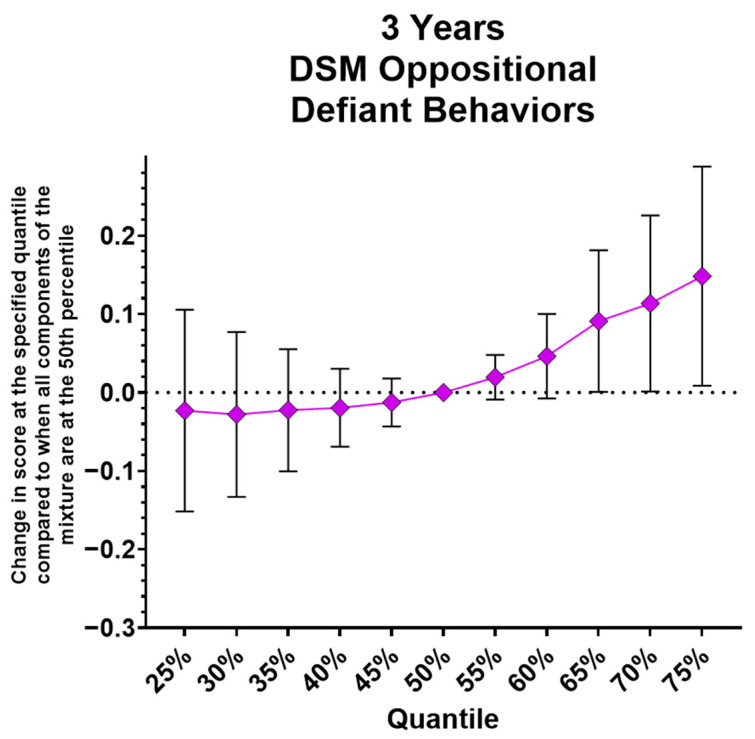
Bayesian Kernel Machine Regression results at 3 years, demonstrating a significant association between the paraben mixture and oppositional defiant problems.

In the analysis of a mixture of the three most commonly detected parabens (ethyl-, methyl-, and propylparaben), two methods were used: Bayesian Kernel Machine Regression (BKMR) and Quantile g-computation. Associations with total problems, internalizing behavior, and externalizing behavior scores at all three ages were examined, as well as subscales associated with individual parabens (age 2: withdrawn, attention problems, and DSM ADHD problems; age 3: aggressive behavior and DSM oppositional defiant problems; age 4: anxious/depressed, somatic complaints, withdrawn, DSM anxiety problems, and DSM pervasive developmental problems). In BKMR analyses, total problems scores were not associated with the mixture at any age. Internalizing behavior scores were only associated with the mixture at 4 years, showing a positive association with the mixture (estimated change in score when all components of the mixture are at their median values [50th percentile] compared to when they are all at their 75th percentile values = 0.055; 95% CI = −0.082, 0.193). No posterior inclusion probabilities (PIPs) were >0.50, but methylparaben was the most important component of the mixture in this relationship (see Table 6). Externalizing behavior scores were positively associated with the mixture at age 2 (estimated change in score when all components of the mixture are at their median values compared to when they are all at their 75th percentile values = 0.085; 95% CI = −0.035, 0.205), and PIPs indicated that ethylparaben was most important for the relationship (see Table 6). At age 2, only DSM ADHD problems scores were positively associated with the mixture (estimated change in score when all components of the mixture are at their median values compared to when they are all at their 75th percentile values = 0.123; 95% CI = −0.004, 0.250), and ethylparaben was the most important component for this relationship (see Table 6). At age 3, only DSM oppositional defiant scores were positively associated with the mixture (estimated change in score when all components of the mixture are at their median values compared to when they are all at their 75th percentile values = 0.148; 95% CI = 0.009, 0.288), and ethylparaben was the most important component in this relationship (see Table 6, Figure 5). At age 4, only anxious/depressed scores were positively associated with the mixture (estimated change in score when all components of the mixture are at their median values compared to when they are all at their 75th percentile values = 0.118; 95% CI = −0.041, 0.276), and methylparaben was the most important component of the mixture for this relationship (see Table 6).

**Figure 6:**
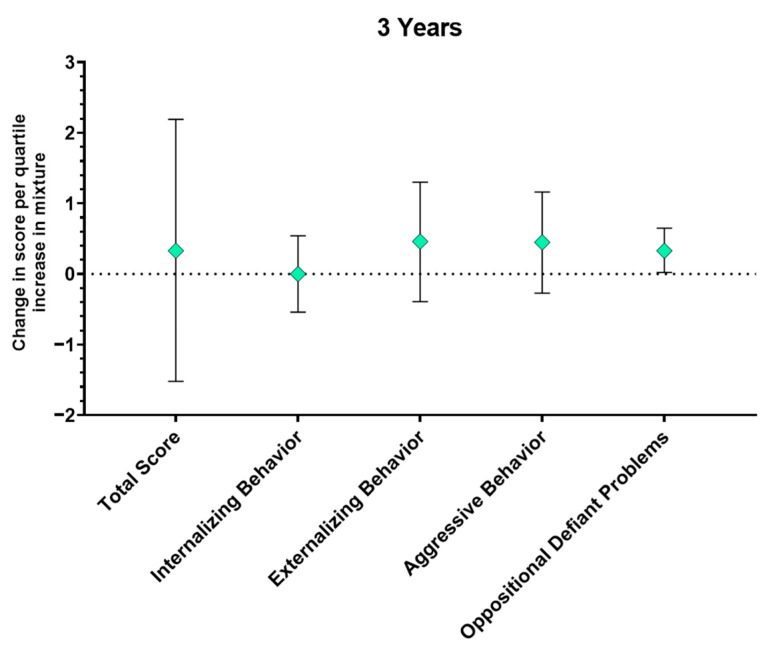
Quantile g-computation results at 3 years, demonstrating a significant association between the paraben mixture and oppositional defiant problems.

In quantile g-computation, few associations with the mixture approached significance. At age 2, the association with the mixture indicated there was a small decrease in withdrawn scores per quartile increase in the mixture (mean change per quartile increase = −0.09; 95% CI = −0.23, 0.05; Table 7), with methylparaben having the largest negative weight (see Table 8). At age 3, there was an increase in oppositional defiant scores per quartile increase in the mixture (mean change per quartile increase = 0.33; 95% CI = 0.02, 0.65), and ethylparaben had the largest positive weight for this association. The mixture was associated with a small increase in anxious/depressed behavior scores at age 4 (mean change per quartile increase = 0.18; 95% CI = −0.10, 0.45), with methylparaben having the largest positive weight and ethylparaben having the largest negative weight. Quantile g-computation provided valuable context to the results seen in the BKMR analysis vis-à-vis the specific directions of association for the component variables. With only one exception (a negative association between ethylparaben and anxious/depressed behavior at age 4), ethylparaben and methylparaben showed association with higher CBCL outcome scores across the board, whereas propylparaben was negatively associated with outcomes in each of the models identified as significant through BKMR, indicative of the latter paraben contributing to lower rather than higher outcome scores (see Table 7 and Table 8, Figure 6).

## 4. Discussion

### 4.1. Key Findings

In general, externalizing behaviors appeared to be most strongly associated with gestational paraben exposure, particularly for males. This was particularly true of exposure to ethylparaben, which was associated with multiple subscales of externalizing behavior at ages 2 and 3. One particular subscale measuring withdrawn symptoms was particularly prominent in the consistency of significant associations we witnessed with paraben exposures. Reduction of withdrawn symptoms in males was associated with each of the three parabens at age two. Differences in associations between time points may be attributable to differences in the subset of children included at each time point or possibly due to developmental trajectories resulting in different behaviors being more sensitive to disruption at different ages. Withdrawn, as a scale, is generally associated with internalizing symptoms, with higher scores indicating less outwardly socializing behavior and lower scores indicating greater outwardly socializing behavior; thus, it is consistent that children who exhibit more externalizing CBCL behaviors, such as ADHD symptoms and oppositional defiant behaviors, would also show lower withdrawn behaviors.

In mixture analysis, the combination of these three parabens demonstrated some trends but only one significant association at one time point (greater oppositional defiant problems at age 3). As discussed in greater detail below, there was a divergence in the direction of impact between the parabens, with propylparaben pushing the mixtures’ associations uniformly in a negative direction, while ethylparaben and methylparaben drove the associations in a positive direction. This may have contributed to the paucity of significant associations relative to what we observed with individual paraben models.

Prior research on prenatal and early childhood paraben exposure as it relates to neurodevelopment is relatively sparse, and very few studies have investigated the possibility of sex-specific associations. Our findings concerning externalizing versus internalizing behaviors contrast those of Skarha et al. (2020) [40], who found no association between a sum of parabens, including butyl-, ethyl-, methyl-, and propylparaben, and either internalizing, externalizing, or the overall behavioral symptoms index using the Behavior Assessment System for Children-2 (BASC-2). However, their study used a different behavioral scale and did not break down the possible associations to individual paraben compounds or individual behavioral subscales, and, as we have demonstrated, the individual parabens demonstrate different patterns of association with different CBCL subscales.

### 4.2. Sex Specificity

Throughout the analyses, males and females have demonstrated different directions of association. Broadly speaking, paraben exposure tended to be associated with more behavior problems in males, whereas fewer significant associations were seen for females, and they were generally in the opposite direction, indicating fewer behavioral problems. This pattern of difference between male and female participants was clearest in CBCL associations with methylparaben, but can be seen as well in the associations of many of the individual CBCL sub-scales with ethyl- and propylparaben as well. Parabens have been established as endocrine disruptors in animal studies and are both weakly estrogenic [41,42] and anti-androgenic [43]. In human studies, prenatal exposure to parabens has been associated with changes in anogenital distance (AGD), a sexually dimorphic, steroid-sensitive developmental marker [44], with higher paraben exposure associated with longer AGD in females and shorter AGD in males. The brain also develops in a sexually dimorphic pattern in response to prenatal steroid hormone exposure, so sex differences in associations between prenatal paraben exposure and behavioral outcomes are not unexpected. Parabens have also been associated with increased markers of oxidative stress and inflammation in pre-partum women, which could represent additional biological mechanisms through which they may impact neurodevelopment [17,45,46,47].

Interestingly, in contrast with the findings of this study, in which female infants were notably less susceptible to changes in CBCL scores associated with paraben exposure, Jiang et al. (2019) [17] found methylparaben and a combination of five parabens to be associated with lower mental development index (MDI) scores, but this significant association was limited to female infants. This difference could be suggestive of the domain specificity of paraben associations, as the MDI scale focuses on cognition, and the CBCL focuses on behavior. Further research would be required to tease out the mechanisms and pathways for this divergence in findings.

### 4.3. Mixture Analyses

The results produced by the two forms of mixture analysis, BKMR and quantile g-computation, are complementary and provide a more comprehensive picture of the associations between gestational paraben exposure and CBCL behavioral outcomes. Of all the mixture analyses conducted, only one identified a significant association in both methods of analysis: that between the paraben mixture and oppositional defiant behaviors at age 3 (See Figure 4 and Figure 5). Based on the results from the quantile g-computation analysis, however, we observe that there are some contradictory forces at play in each of the associations; propylparaben tends to pull those associations in the opposite direction from ethyl- and methylparaben, thereby weakening the strength of the overall association.

The mixture models also demonstrated an interesting pattern of difference between propylparaben on the one hand, and ethyl- and methylparaben on the other. In mixture models, the direction of association for propylparaben was, in all cases, negative, balancing against the almost entirely positive directions of association for ethyl- and methylparaben; the only exceptions to the latter pattern being one negative association between methylparaben and withdrawn behaviors at age 2, and one between ethylparaben and anxious/depressed behavior at age 4. To some extent, this pattern was demonstrated in the individual paraben associations as well. Propylparaben consistently showed a negative association with CBCL outcomes (e.g., fewer behavior problems), particularly at 2 and 3 years of age, and particularly in females. At these first two time points, ethylparaben and methylparaben generally showed a mixture of positive and negative associations, but the statistically significant ones were uniformly in the positive direction (more behavior problems).

Although many of these associations did not cross the threshold for statistical significance, a clear enough pattern of difference has emerged that it is worth remarking upon. Throughout the analyses, particularly at the younger ages and for externalizing behavior in males—notably the two criteria most frequently associated with significant relationships—propylparaben demonstrates directions of association opposed to those of ethylparaben and methylparaben, to the extent that its inclusion in our mixtures analyses may be dampening the effect size and significance of the two other paraben biomarkers. This may help to explain the relative dearth of significant associations observed in the mixture analyses relative to the individual chemical regression models. Patterns in the directions of association between these three parabens have been mixed in previous studies, but in at least one study [48], propylparaben was consistently shown to have opposing relationships to neurodevelopmental scales on the Mullen Scales of Early Learning at age 3 from those of methylparaben. The differences in patterns of association identified here highlight the importance of assessing and comparing multiple paraben compounds rather than a single compound or a sum of parabens alone.

### 4.4. Strengths and Limitations

This study has several strengths. First, it employed multiple methods of mixture analysis, which is valuable due to Bayesian Kernel Machine Regression (BKMR) being more sensitive to non-normal data distributions in the exposures and providing posterior inclusion probabilities to identify which components of the mixture had the strongest impact on the strength of the association, while Quantile g-computation provides both effect sizes and directions of association. Together, these approaches complement one another, providing a richer context for the overall analysis. Second, the study incorporated individual chemical analyses, which are consistent with established methods in prior research, and combined them with mixture analyses that are more reflective of real-world exposure patterns and lived experiences. Third, the longitudinal follow-up of children with behavioral assessment in this study strengthens temporal inference. Lastly, the use of a standardized and validated instrument (CBCL) enhances the reliability of childhood behavioral assessments.

This study also has limitations. First, the relatively small, homogeneous sample may reduce the power and generalizability of the study findings. The size of the sample was also such that we were unable to consider the impact of butylparaben due to its relatively low detection rate in the sample (<50%). Moreover, this being a cohort study, we did see some significant attrition in the sample, as only 68.4% of participants who completed the CBCL checklist at age 2 eventually completed it at age 4. Because the subsamples at each of the three time points were overlapping but not identical, and the sample size was relatively small, we elected to employ a cross-sectional analysis at each time point rather than a longitudinal model. Lastly, this study only considers the associations of prenatal paraben exposure with infant neurodevelopment and does not address the potential impacts of postnatal exposure.

Despite these limitations, the findings of this study suggest that the impact of prenatal exposure to parabens on early childhood behavior should be further investigated. For studies with larger and more diverse samples, one important avenue of inquiry might be to investigate the role of paraben exposure in potentially exacerbating existing disparities between populations differently affected by paraben exposure. Studies using CBCL data have identified disparities in scores across social groups, including along lines of race, ethnicity, and socioeconomic status, reflective of disparities in education, access to nutritious diet, exposure to stress, and, most saliently, exposure to harmful chemicals, including parabens [20,49].

## 5. Conclusions

The findings in this study suggest that gestational paraben exposure may lead to changes in early behavioral development. Males seem especially susceptible to behavioral associations with exposure, associations that appear primarily linked to externalizing behaviors. Behaviors in female children do not appear to be as strongly associated with the gestational paraben exposures, although to the extent that we do see significant links, the patterns of association are markedly different from those seen in males, with associations frequently indicating lower rather than higher CBCL scores for females with higher gestational paraben exposures. We have also demonstrated that propylparaben is often associated with opposite directions of association from ethyl- and methylparaben, in mixture analysis, affecting a negative push on associations with CBCL outcomes, while the other two parabens push the association in a positive direction.

The results here suggest that concern may be warranted regarding exposure of developing fetuses to gestational parabens, and further research is called for to establish both the mechanisms by which gestational paraben exposure could impact early child behavior and the interactions that gestational exposure may have with other environmental risk factors.

## Figures and Tables

**Table 1:** Categorical parental demographics for all IKIDS participants with exposure data available and an infant enrolled at birth and each subsample who provided CBCL data when children were 2, 3, and 4 years. Adapted from Woodbury et al. (2024) Table 1 [33].

Parental Demographics	Participants with Exposure Data	Participants with Exposure and CBCL Data at 2 Years	Participants with Exposure and CBCL Data at 3 Years	Participants with Exposure and CBCL Data at 4 Years	
	(*n* = 514)	(*n* = 285)	(*n* = 255)	(*n* = 195)	
	N (%)	N (%)	N (%)	N (%)	*p*-Value
**Maternal race & ethnicity**					0.69
White, Non-Hispanic	428 (83.3)	244 (85.6)	222 (87.1)	171 (87.7)	
Other	84 (16.3)	41 (14.4)	33 (12.9)	24 (12.3)	
Unknown/Missing	2 (0.4)	0 (0.0)	0 (0,0)	0 (0.0)	
**Maternal marital status**					0.31
Married/Living as married	476 (92.6)	270 (94.7)	244 (95.7)	185 (94.9)	
Separated/Divorced/Widowed/Single	38 (7.4)	15 (5.3)	11 (4.3)	10 (5.1)	
Unknown/Missing	0 (0.0)	0 (0.0)	0 (0.0)	0 (0.0)	
**Maternal education**					0.003
<Bachelor’s degree	97 (18.9)	34 (11.9)	27 (10.6)	25 (12.8)	
≥Bachelor’s degree	417 (81.1)	251 (88.1)	228 (89.4)	170 (87.2)	
**Maternal parity**					0.76
0	262 (51.0)	158 (55.4)	134 (52.5)	105 (53.9)	
≥1	251 (48.8)	127 (44.6)	121 (47.5)	90 (46.1)	
Missing	1 (0.2)	0 (0.0)	0 (0.0)	0 (0.0)	
**Household income**					0.33
$0–$49,999	96 (18.7)	46 (16.1)	35 (13.7)	27 (13.8)	
$50,000–$99,999	248 (48.2)	129 (45.3)	127 (49.8)	98 (50.3)	
≥$100,000	166 (32.3)	110 (38.6)	93 (36.5)	70 (35.9)	
Unknown/Missing	4 (0.8)	0 (0.0)	0 (0.0)	0 (0.0)	
**Prenatal tobacco use**					0.46
Yes	26 (5.1)	8 (2.8)	10 (3.9)	6 (3.1)	
No	488 (94.4)	277 (97.2)	245 (96.1)	189 (96.9)	
Unknown/Missing	0 (0.0)	0 (0.0)	0 (0.0)	0 (0.0)	
**Prenatal alcohol use during whole pregnancy**					0.95
Yes	387 (75.3)	211 (74.0)	195 (76.5)	145 (74.4)	
No	127 (24.7)	74 (26.0)	60 (23.5)	50 (25.6)	

**Table 2:** Continuous parental demographics for all IKIDS participants with exposure data available and an infant enrolled at birth and each subsample who provided CBCL data when children were 2, 3, and 4 years. Adapted from Woodbury et al. (2024) Table 2 [33].

Parental Demographics	Participants with Exposure Data	Participants with Exposure and CBCL Data at 2 Years	Participants with Exposure and CBCL Data at 3 Years	Participants with Exposure and CBCL Data at 4 Years	
	(*n* = 514)	(*n* = 285)	(*n* = 255)	(*n* = 195)	*p*-Value
	Mean (SD)	Mean (SD)	Mean (SD)	Mean (SD)	
**Maternal age (years) at baseline**	30.33 (4.11)	30.70 (3.83)	30.69 (3.72)	30.53 (3.69)	0.73
**Maternal verbal IQ**	107.88 (11.36)	108.32 (11.19)	109.42 (10.86)	109.45 (10.56)	0.34
**(PPVT ^a^ standardized score)**					
**Mean maternal stress (PSS-10) ** **^b^** ** score during child’s infancy**	10.32 (6.11)	10.19 (5.93)	9.96 (5.99)	9.89 (5.91)	0.75
**Mean maternal depression (EPDS) ** **^c^** ** score during child’s infancy**	3.85 (3.47)	3.81 (3.28)	3.57 (3.29)	3.61 (3.22)	0.76

^a^ PPVT-IV: Peabody Picture Vocabulary Test—Fourth Edition, ^b^ PSS-10: Perceived Stress Scale, ^c^ EPDS: Edinburgh Postnatal Depression Scale.

**Table 3:** Categorical child demographics for all IKIDS participants with exposure data available and an infant enrolled at birth and each subsample who provided CBCL data when children were 2, 3, and 4 years.

Child Demographics	Participants with Exposure Data	Participants with Exposure and CBCL Data at 2 Years	Participants with Exposure and CBCL Data at 3 Years	Participants with Exposure and CBCL Data at 4 Years	
	(*n* = 514)	(*n* = 285)	(*n* = 240)	(*n* = 195)	*p*-Value
	N (%)	N (%)	N (%)	N (%)	
**Child sex**					0.94
Male	252 (49.0)	136 (47.7)	126 (49.4)	98 (50.3)	
Female	262 (51.0)	149 (52.3)	129 (50.6)	97 (49.7)	
**Child race & ethnicity**					0.45
White, Non-Hispanic	387 (75.3)	229 (80.4)	207 (81.2)	157 (80.5)	
Other	125 (24.3)	56 (19.6)	48 (18.8)	38 (19.5)	
Unknown/Missing	2 (0.4)	0 (0.0)	0 (0.0)	0 (0.0)	
**Delivery type**					0.81
Vaginal	138 (26.8)	206 (72.3)	183 (71.8)	136 (69.7)	
Cesarean section	357 (69.5)	70 (24.6)	63 (24.7)	51 (26.2)	
Unknown/Missing	19 (3.7)	9 (3.1)	9 (3.5)	8 (4.1)	

*p*-values for categorical variables estimated using Chi-square tests, Wilcoxon-sum rank tests for continuous variables (no differences in pairwise comparisons).

**Table 4:** Maternal urinary biomarker characterization for IKIDS infants included in the current analysis compared to samples collected from females in NHANES between 2015 and 2016.

	NHANES Females (2015–2016)	All IKIDS Participants		IKIDS Participants Included at 2 Years		IKIDS Participants Included at 3 Years		IKIDS Participants Included at 4 Years		
		*n* = 514		*n* = 285		*n* = 255		*n* = 195		
Paraben Metabolite	Median (95% CI)	Median (IQR)	N (%) < LOD	Median (IQR)	N (%) < LOD	Median (IQR)	N (%) < LOD	Median (IQR)	N (%) < LOD	
	(μg/L)	(μg/L)		(μg/L)		(μg/L)		(μg/L)		*p*-Value
EtPB	1.30 (<LOD-2.00)	1.20 (6.26)	7 (1.4)	1.30 (5.45)	4 (1.4)	1.10 (5.35)	3 (1.2)	1.00 (3.10)	3 (1.5)	0.57
MePB	59.20 (40.80–86.50)	48.45 (113.50)	0 (0.0)	50.40 (113.40)	0 (0.0)	46.20 (101.40)	0 (0.0)	40.50 (99.20)	0 (0.0)	0.50
PrPB	8.90 (5.60–14.10)	8.10 (25.80)	0 (0.0)	8.00 (24.10)	0 (0.0)	7.10 (23.90)	0 (0.0)	6.20 (19.40)	0 (0.0)	0.51

*p*-values estimated using Wilcoxon-sum rank tests (no differences among groups in pairwise comparisons), National Center for Environmental Health. National Report on Human Exposure to Environmental Chemicals. U.S. Department of Health and Human Services, Centers for Disease Control and Prevention. 2022. Updated September 2023. Accessed 7 April 2024. https://doi.org/10.15620/cdc:133100.

**Table 5:** Continuous child demographics for all IKIDS participants with exposure data available and an infant enrolled at birth and each subsample who provided CBCL data when children were 2, 3, and 4 years.

Child Demographics	Participants with Exposure Data	Participants with Exposure and CBCL Data at 2 Years	Participants with Exposure and CBCL Data at 3 Years	Participants with Exposure and CBCL Data at 4 Years	*p*-Value
	Mean (SD)	Mean (SD)	Mean (SD)	Mean (SD)	
**Child gestational age at birth (weeks)**	39.34 (1.41)	39.39 (1.51)	39.42 (1.51)	39.46 (1.47)	0.38
**Child weight at birth (kg)**	3.48 (0.43)	3.50 (0.43)	3.51 (0.43)	3.54 (0.44)	0.55
**Child age at time CBCL was completed (months)**	--	27.94 (1.01)	37.51 (0.93)	48.10 (1.11)	

**Table 6:** Posterior inclusion probabilities (PIPs) for conditional inclusion in models of the effect of the gestational paraben mixture on select CBCL outcomes estimated using Bayesian Kernel Regression (BKMR).

	CBCL Completed at 2 Years								
Paraben	Total Score	Internalizing Problems	Withdrawn Behavior	Anxious/ Depressed Behavior	Externalizing Problems	Attention Problems	ADHD Problems	Aggressive Behavior	Oppositional Defiant Problems
EtPB	0.62698	0.61008	0.61222		0.63328	0.7453	0.91292		
MePB	0.61572	0.61568	0.6262		0.35868	0.59494	0.47628		
PrPB	0.61038	0.61742	0.61954		0.35854	0.51872	0.37198		
	**CBCL completed at 3 years**								
EtPB	0.64046	0.52088			0.67252			0.7453	0.91292
MePB	0.62552	0.53212			0.33164			0.59494	0.47628
PrPB	0.62992	0.53884			0.31174			0.51872	0.37198
	**CBCL completed at 4 years**								
EtPB	0.26428	0.24712		0.26686	0.18802				
MePB	0.28028	0.31828		0.57128	0.23228				
PrPB	0.27086	0.26236		0.36238	0.1851				

**Table 7:** Quantile g-computation estimates and 95% confidence intervals for the change in select CBCL outcomes per one quartile increase in the paraben mixture.

	CBCL Completed at 2 Years	CBCL Completed at 3 Years	CBCL Completed at 4 Years
CBCL Outcome	β (95% CI)	β (95% CI)	β (95% CI)
Total Score	0.96 (−0.75, 2.67)	0.33 (−1.52, 2.19)	0.78 (−1.65, 3.20)
Internalizing Problems	−0.09 (−0.55, 0.37)	0 (−0.54, 0.54)	0.30 (−0.46, 1.06)
Withdrawn Behavior	−0.09 (−0.23, 0.05)	--	--
Anxious/Depressed Behavior	--	--	0.18 (−0.10, 0.45)
Externalizing Problems	0.47 (−0.32, 1.27)	0.46 (−0.39, 1.30)	0.67 (−0.33, 1.68)
Attention Problems	0.01 (−0.21, 0.22)	--	--
Aggressive Behavior	--	0.45 (−0.27, 1.16)	--
ADHD Problems	0.15 (−0.14, 0.45)	--	--
Oppositional Defiant Problems	--	0.33 (0.02, 0.65)	--

**Table 8:** Relative positive (+) and negative (−) weights estimated from quantile g-computation for each paraben in the mixture for select CBCL outcomes.

	CBCL Completed at 2 Years																	
Paraben	Total Score		Internalizing Problems		Withdrawn Behavior		Anxious/ Depressed Behavior		Externalizing Problems		Attention Problems		ADHD Problems		Aggressive Behavior		Oppositional Defiant Problems	
EtPB	+	0.43	+	0.26	+	1.00			+	0.45	+	0.76	+	0.67				
MePB	+	0.57	+	0.74	−	0.88			+	0.55	+	0.24	+	0.33				
PrPB	−	1.00	−	1.00	−	0.12			−	1.00	−	1.00	−	1.00				
	**CBCL completed at 3 years**																	
EtPB	+	0.69	+	0.54					+	0.55					+	0.57	+	0.87
MePB	+	0.31	+	0.46					+	0.45					+	0.43	+	0.13
PrPB	−	1.00	−	1.00					−	1.00					−	1.00	−	1.00
	**CBCL completed at 4 years**																	
EtPB	+	0.55	+	0.44			−	0.61	+	0.48								
MePB	+	0.45	+	0.56			+	1.00	+	0.52								
PrPB	−	1.00	−	1.00			−	0.39	−	1.00								

## Data Availability

The de-identified data presented in the study are openly available in the Data and Specimen Hub (DASH) maintained by the National Institute of Child Health and Hu-man Development (NICHD) at https://dash.nichd.nih.gov/.

## Supplementary Materials

The following supporting information can be downloaded at: https://www.mdpi.com/article/10.3390/toxics14030211/s1, Table S1: Distributions of CBCL scores at 2, 3, and 4 years in IKIDS for all children and comparisons of male and female children; Figure S1: Directed acyclic graph of hypothesized associations between gestational paraben exposures, CBCL behavioral outcomes, and potential confounder variables; Table S2: Categorical parental demographics for all IKIDS participants with exposure data available and an infant enrolled at birth and each subsample who provided CBCL data when children were 2, 3, and 4 years; Table S3: Continuous parental demographics for all IKIDS participants with exposure data available and an infant enrolled at birth and each subsample who provided CBCL data when children were 2, 3, and 4 years; Table S4: Categorical child demographics for all IKIDS participants with exposure data available and an infant enrolled at birth and each subsample who provided CBCL data when children were 2, 3, and 4 years; Figure S2: Bayesian Kernel Machine Regression results for the total problems scores (top row), internalizing problems scores (middle row), and externalizing problems scores (bottom row) at ages 2 (left column), 3 (middle column), and 4 (right column). The parabens mixture was not associated with the total problems score at age 2 (A), 3 (B), or 4 (C). It was also not associated with internalizing problems scores at 2 (D) or 3 years of age (E), but there was a positive association at age 4 (F). The mixture was also positively associated with externalizing problems scores at age 2 (G), but not at ages 3 (H) or 4 (I); Figure S3: Bayesian Kernel Machine Regression results for the withdrawn behavior (A), attention problems (B), and DSM ADHD behaviors (C) subscales at age 2. The mixture was not associated with (A) withdrawn behavior or (B) attention problems scores, but it was positively associated with (C) DSM ADHD behaviors scores; Figure S4: Bayesian Kernel Machine Regression results for the aggressive problems subscale at age 3. There was no clear association of the mixture with aggressive problems scores; Figure S5: Bayesian Kernel Machine Regression results for the anxious/depressed problems (A), somatic complaints (B), withdrawn behavior (C), pervasive developmental problems (D), and DSM anxiety problems (E) subscale scores at age 4. Only anxious/depressed problems were associated with the mixture at 4 years of age; Table S5: Sensitivity analysis: Multivariable linear regression analyses of the relation (β estimate and 95% confidence interval) of gestational paraben exposure with CBCL scores at ages 2, 3, and 4 years with any maternal alcohol use during pregnancy included in models; Table S6: Sensitivity analysis: Multivariable linear regression analyses of the relation (β estimate and 95% confidence interval) of gestational paraben exposure with CBCL scores at ages 2, 3, and 4 years with mothers who smoked during pregnancy excluded; Table S7: Sensitivity analysis: Multivariable linear regression analyses of the relation (β estimate and 95% confidence interval) of gestational paraben exposure with CBCL scores at ages 2, 3, and 4 years with influential cases (identified with a Cook’s distance > 0.06) excluded; Table S8: Multivariable linear regression analyses of the relation (β estimate and 95% confidence interval) of gestational paraben exposure with CBCL scores at 2 years of age, stratified; Table S9: Multivariable linear regression analyses of the relation (β estimate and 95% confidence interval) of gestational paraben exposure with CBCL scores at 3 years of age, stratified by sex; Table S10: Multivariable linear regression analyses of the relation (β estimate and 95% confidence interval) of gestational paraben exposure with CBCL scores at 4 years of age, stratified by sex.

## Abbreviations

The following abbreviations are used in this manuscript:

CBCL	Child Behavior Checklist
IKIDS	Illinois Kids Development Study
ADHD	Attention Deficit Hyperactivity Disorder
PSS	Perceived Stress Scale
EPDS	Edinburgh Postnatal Depression Scale
BKMR	Bayesian Kernel Machine Regression
DSM	Diagnostic and Statistical Manual of Mental Disorders
PIPs	Posterior inclusion probabilities
NHANES	National Health and Nutrition Examination Survey
