# Supplementary material for: Associations of Exposure to Parabens During Pregnancy with Behavior in Early Childhood
Source: Toxics. 2026 Feb 28;14(3):211. doi: 10.3390/toxics14030211 (PMC13030551; doi:10.3390/toxics14030211)
Supplement: Supplementary file 1 [file toxics-14-00211-s001.zip › toxics-4028371-supplementary.pdf]

Associations of exposure to parabens during pregnancy with behavior in early childhood

Megan L. Woodbury,[1] Nicholas Cragoe,[2] Susan L. Schantz[3]
Corresponding author: Nicholas Cragoe, PhD

Cragoe2@illinois.edu, 612-418-1682
405 N. Mathews Ave.
Beckman Institute for Advanced Science and Technology, University of Illinois Urbana-Champaign

[1]PROTECT Center, College of Engineering, Northeastern University, Boston, MA. Department of Communication Sciences and Disorders, Northeastern University, Boston, MA.
[2]Beckman Institute for Advanced Science and Technology, University of Illinois at Urbana-Champaign, Urbana, IL.
[3] Beckman Institute for Advanced Science and Technology, University of Illinois at Urbana-Champaign, Urbana, IL. Department of Comparative Biosciences, University of Illinois at Urbana-Champaign, Urbana, IL.

Supplemental Table 1. Distributions of CBCL scores at 2, 3, and 4 years in IKIDS for all children and comparisons of male and female children.

Outcome measures				Females			Males			p-value
CBCL (2 year)	n	Mean ± SEM	Median	n	Mean ± SEM	Median	n	Mean ± SEM	Median	
Total Problems Score	260	20.56 ± 0.85	18.00	138	20.01 ± 1.09	19.00	122	21.18 ± 1.33	17.50	0.49
Internalizing Problems	269	4.11 ± 0.22	3.00	141	4.06 ± 0.30	3.00	128	4.16 ± 0.33	3.00	0.81
Emotionally Reactive	277	1.27 ± 0.09	1.00	145	1.24 ± 0.12	1.00	132	1.31 ± 0.13	1.00	0.70
Anxious/Depressed	277	1.15 ± 0.08	1.00	145	1.21 ± 0.11	1.00	132	1.08 ± 0.12	1.00	0.44
Somatic Complaints	278	1.07 ± 0.08	1.00	145	1.05 ± 0.11	1.00	133	1.10 ± 0.11	1.00	0.79
Withdrawn	274	0.69 ± 0.07	0.00	144	0.65 ± 0.08	0.00	130	0.73 ± 0.11	0.00	0.56
Pervasive Dev. Probs.	271	1.86 ± 0.12	1.00	143	1.72 ± 0.16	1.00	128	2.01 ± 0.18	1.00	0.25
Anxiety Problems	275	1.69 ± 0.10	1.00	144	1.73 ± 0.13	2.00	131	1.64 ± 0.16	1.00	0.67
Affective Problems	277	1.19 ± 0.09	3.00	144	1.10 ± 0.11	1.00	133	1.29 ± 0.14	1.00	0.29
Sleep Problems	277	1.88 ± 0.13	1.00	145	1.83 ± 0.16	1.00	132	1.93 ± 0.20	1.00	0.68
Externalizing Problems	270	9.12 ± 0.39	8.00	143	8.92 ± 0.52	8.00	127	9.34 ± 0.58	8.00	0.59
Attention Problems	274	1.75 ± 0.10	1.00	145	1.75 ± 0.13	1.00	129	1.76 ± 0.16	1.00	0.97
Aggressive Behavior	275	7.36 ± 0.32	7.00	144	7.17 ± 0.44	7.50	131	7.56 ± 0.46	7.00	0.55
ADHD Problems	277	3.57 ± 0.14	3.00	147	3.43 ± 0.18	3.00	130	3.72 ± 0.22	4.00	0.32
Oppos. Defiant Probs.	277	2.22 ± 0.13	2.00	145	2.75 ± 0.18	3.00	132	2.76 ± 0.20	2.00	0.98
CBCL (3 years)										
Total Problems Score	235	19.78 ± 0.93	17.00	114	17.97 ± 1.29	14.50	121	21.48 ± 1.31	19.00	0.06
Internalizing Problems	246	4.52 ± 0.26	3.00	122	4.44 ± 0.38	3.00	124	4.60 ± 0.37	4.00	0.77
Emotionally Reactive	250	1.63 ± 0.11	1.00	125	1.58 ± 0.16	1.00	125	1.69 ± 0.14	1.00	0.60
Anxious/Depressed	254	1.08 ± 0.09	0.00	127	1.19 ± 0.14	1.00	127	0.98 ± 0.12	0.00	0.26
Somatic Complaints	253	1.18 ± 0.09	1.00	127	1.11 ± 0.11	1.00	126	1.25 ± 0.14	1.00	0.45
Withdrawn	255	0.59 ± 0.07	0.00	128	0.54 ± 0.08	0.00	127	0.64 ± 0.11	0.00	0.42
Pervasive Dev. Probs.	251	1.78 ± 0.12	1.00	127	1.55 ± 0.14	1.00	124	2.02 ± 0.19	2.00	**0.05**
Anxiety Problems	254	1.89 ± 0.11	1.00	127	1.87 ± 0.17	1.00	127	1.92 ± 0.16	2.00	0.81
Affective Problems	252	1.67 ± 0.09	1.00	125	1.11 ± 0.12	1.00	127	1.22 ± 0.13	1.00	0.54
Sleep Problems	252	2.23 ± 0.14	2.00	125	2.14 ± 0.19	1.00	127	2.33 ± 0.20	2.00	0.48
Externalizing Problems	245	8.14 ± 0.41	7.00	121	7.33 ± 0.55	6.00	124	8.93 ± 0.60	8.00	**0.05**
Attention Problems	254	1.42 ± 0.09	1.00	128	1.22 ± 0.11	1.00	126	1.62 ± 0.15	1.00	**0.04**
Aggressive Behavior	247	6.69 ± 0.35	6.00	122	6.11 ± 0.47	4.00	125	7.26 ± 0.50	7.00	0.10
ADHD Problems	252	2.91 ± 0.14	3.00	126	2.55 ± 0.18	2.00	126	3.28 ± 0.21	3.00	**0.01**
Oppos. Defiant Probs.	252	2.67 ± 0.15	2.00	126	2.48 ± 0.20	2.00	126	2.86 ± 0.21	2.00	0.21
CBCL (4 years)										
Total Problems Score	176	21.55 ± 1.17	19.00	85	19.90 ± 1.58	18.00	91	23.09 ± 1.71	21.00	0.17
Internalizing Problems	185	5.06 ± 0.35	4.00	90	4.94 ± 0.50	4.00	95	5.18 ± 0.50	4.00	0.74
Emotionally Reactive	194	1.78 ± 0.14	1.00	96	1.72 ± 0.18	1.00	98	1.84 ± 0.20	1.00	0.66
Anxious/Depressed	194	1.35 ± 0.12	1.00	96	1.22 ± 0.16	1.00	98	1.49 ± 0.19	1.00	0.28
Somatic Complaints	191	1.28 ± 0.11	1.00	94	1.36 ± 0.17	1.00	97	1.21 ± 0.14	1.00	0.49
Withdrawn	193	0.72 ± 0.08	0.00	94	0.70 ± 0.11	0.00	99	0.74 ± 0.13	0.00	0.84
Pervasive Dev. Probs.	190	2.21 ± 0.15	2.00	93	2.12 ± 0.21	2.00	97	2.31 ± 0.23	2.00	0.54
Anxiety Problems	193	2.22 ± 0.15	2.00	97	1.97 ± 0.19	2.00	96	2.48 ± 0.24	2.00	0.10
Affective Problems	193	1.45 ± 0.12	1.00	95	1.39 ± 0.17	1.00	98	1.51 ± 0.19	1.00	0.63
Sleep Problems	194	2.57 ± 0.17	2.00	96	2.48 ± 0.24	2.00	98	2.66 ± 0.25	2.00	0.60
Externalizing Problems	187	8.53 ± 0.48	8.00	91	7.04 ± 0.64	5.00	96	9.95 ± 0.70	9.00	**0.002**
Attention Problems	193	1.55 ± 0.11	1.00	94	1.21 ± 0.14	1.00	99	1.87 ± 0.17	2.00	**0.003**

Aggressive Behavior	190	7.02 ± 0.41	6.00	94	5.95 ± 0.56	5.00	96	8.07 ± 0.59	8.00	**0.01**
ADHD Problems	195	3.02 ± 0.17	3.00	97	2.49 ± 0.21	2.00	98	3.54 ± 0.25	3.00	**0.002**
Oppos. Defiant Probs.	195	2.69 ± 0.17	2.00	96	2.25 ± 0.23	2.00	99	3.12 ± 0.24	3.00	**0.01**

Supplemental Figure 1. Directed acyclic graph of hypothesized associations between gestational paraben exposures, CBCL behavioral outcomes, and potential confounder variables.

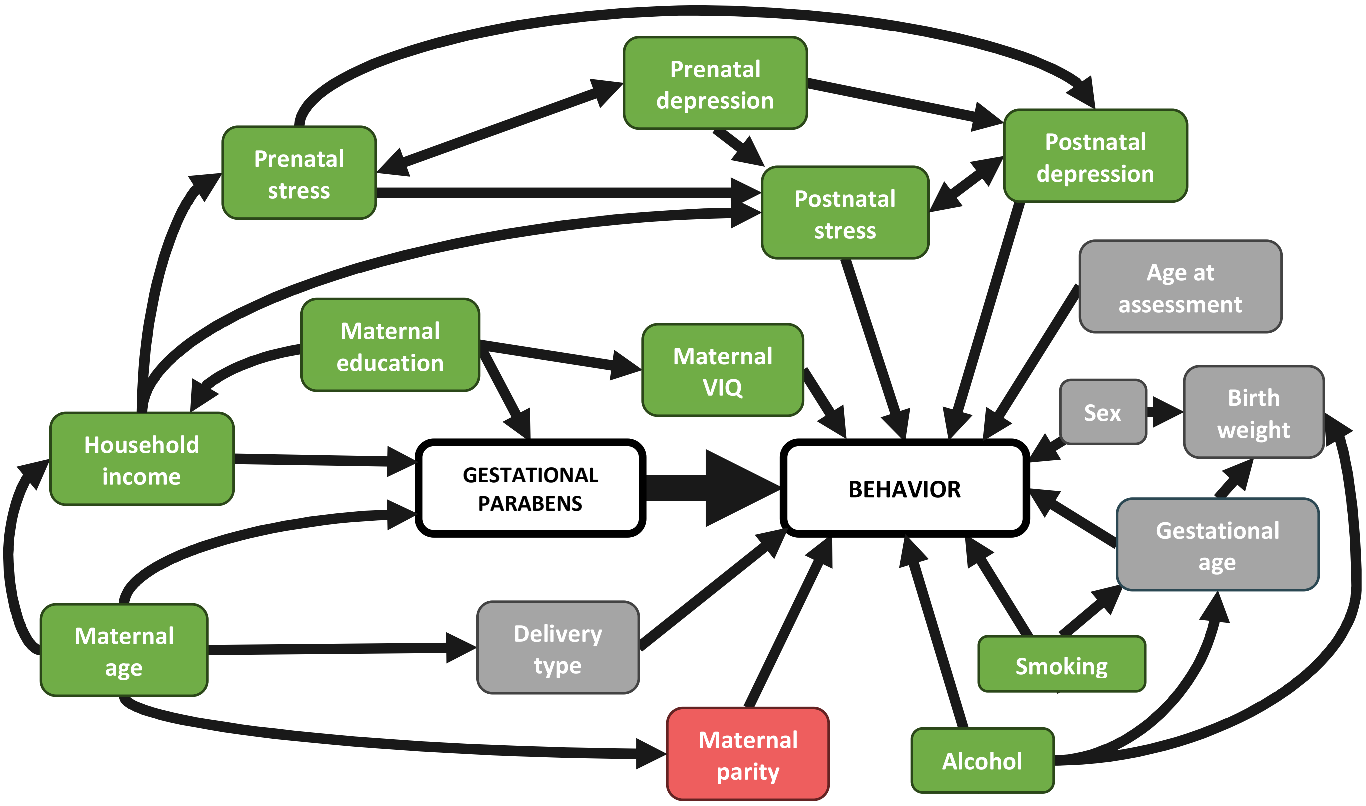

Supplemental Table 2. Categorical parental demographics for all IKIDS participants with exposure data available and an infant enrolled at birth and each subsample who provided CBCL data when children were 2, 3, and 4 years.

**Parental demographics**	**Participants with exposure data** *(n = 514)*	**Participants with exposure and CBCL data at 2 yrs** *(n = 285)*	**Participants with exposure and CBCL data at 3 yrs** *(n = 255)*	**Participants with exposure and CBCL data at 4 yrs** *(n = 195)*	
	**N (%)**	**N (%)**	**N (%)**	**N (%)**	**p-value†**
**Maternal race & ethnicity**					0.69

White, Non-Hispanic	428 (83.3)	244 (85.6)	222 (87.1)	171 (87.7)	
Other	84 (16.3)	41 (14.4)	33 (12.9)	24 (12.3)	
Unknown/Missing	2 (0.4)	0 (0.0)	0 (0,0)	0 (0.0)	
**Paternal race & ethnicity**					0.22
White, Non-Hispanic	418 (81.3)	249 (87.4)	222 (87.1)	170 (87.2)	
Other	94 (18.3)	36 (12.6)	33 (12.9)	25 (12.8)	
Unknown/Missing	2 (0.4)	0 (0.0)	0 (0,0)	0 (0.0)	
**Maternal marital status**					0.31
Married/Living as married	476 (92.6)	270 (94.7)	244 (95.7)	185 (94.9)	
Separated/Divorced/Widowed/ Single	38 (7.4)	15 (5.3)	11 (4.3)	10 (5.1)	
Unknown/Missing	0 (0.0)	0 (0.0)	0 (0.0)	0 (0.0)	
**Maternal education**					**0.003**
< Bachelor's degree	97 (18.9)	34 (11.9)	27 (10.6)	25 (12.8)	
≥ Bachelor's degree	417 (81.1)	251 (88.1)	228 (89.4)	170 (87.2)	
**Paternal education**					0.07
< Bachelor's degree	152 (29.6)	60 (21.1)	56 (22.0)	45 (23.1)	
≥ Bachelor's degree	359 (69.8)	225 (78.9)	199 (78.0)	150 (76.9)	
Unknown/Missing	3 (0.6)	0 (0.0)	0 (0.0)	0 (0.0)	
**Maternal parity**					0.76
0	262 (51.0)	158 (55.4)	134 (52.5)	105 (53.9)	
≥1	251 (48.8)	127 (44.6)	121 (47.5)	90 (46.1)	
Missing	1 (0.2)	0 (0.0)	0 (0.0)	0 (0.0)	
**Household income**					0.33
$0-$49,999	96 (18.7)	46 (16.1)	35 (13.7)	27 (13.8)	
$50,000-$99,999	248 (48.2)	129 (45.3)	127 (49.8)	98 (50.3)	
≥$100,000	166 (32.3)	110 (38.6)	93 (36.5)	70 (35.9)	
Unknown/Missing	4 (0.8)	0 (0.0)	0 (0.0)	0 (0.0)	
**Prenatal tobacco use**					0.46
Yes	26 (5.1)	8 (2.8)	10 (3.9)	6 (3.1)	
No	488 (94.4)	277 (97.2)	245 (96.1)	189 (96.9)	
Unknown/Missing	0 (0.0)	0 (0.0)	0 (0.0)	0 (0.0)	
**Prenatal alcohol use during whole pregnancy**					0.95
Yes	387 (75.3)	211 (74.0)	195 (76.5)	145 (74.4)	
No	127 (24.7)	74 (26.0)	60 (23.5)	50 (25.6)	

Prenatal alcohol use during 1st trimester					
Gestational weeks 0-4					0.92
None	304 (59.1)	172 (60.34)	151 (59.2)	121 (62.0)	
< 4 drinks/week	166 (32.3)	90 (31.6)	77 (30.2)	65 (33.3)	
4-10 drinks/week	37 (7.2)	19 (6.7)	22 (8.6)	16 (8.2)	
≥11 drinks/week	6 (1.2)	4 (1.4)	5 (2.0)	1 (0.5)	
Unknown/Missing	1 (0.2)	0 (0.0)	0 (0.0)	0 (0.0)	
Gestational weeks 5-8					0.87
None	497 (96.7)	275 (96.5)	245 (96.1)	189 (96.9)	
< 4 drinks/week	9 (1.7)	7 (2.4)	4 (1.6)	3 (1.5)	
Unknown/Missing	8 (1.6)	3 (1.0)	6 (2.3)	3 (1.5)	
Gestational weeks 9-14					0.92
None	504 (98.0)	279 (97.9)	251 (98.4)	191 (98.0)	
< 4 drinks/week	6 (1.2)	4 (1.4)	2 (0.8)	2 (1.0)	
Unknown/Missing	4 (0.8)	2 (0.7)	2 (0.8)	2 (1.0)	
[a]PPVT-IV: Peabody Picture Vocabulary Test - Fourth Edition					
[c]PSS-10: Perceived Stress Scale					

Supplemental Table 3. Continuous parental demographics for all IKIDS participants with exposure data available and an infant enrolled at birth and each subsample who provided CBCL data when children were 2, 3, and 4 years.

Parental demographics	Participants with exposure data (n = 514)	Participants with exposure and CBCL data at 2 yrs (n = 285)	Participants with exposure and CBCL data at 3 yrs (n = 255)	Participants with exposure and CBCL data at 4 yrs (n = 195)	p-value†
	Mean (SD)	Mean (SD)	Mean (SD)	Mean (SD)	
**Maternal age (years) at baseline**	30.33 (4.11)	30.70 (3.83)	30.69 (3.72)	30.53 (3.69)	0.73
**Maternal verbal IQ (PPVT[a] standardized score)**	107.88 (11.36)	108.32 (11.19)	109.42 (10.86)	109.45 (10.56)	0.34
**Mean maternal stress (PSS-10[c]) score during pregnancy**	11.18 (5.68)	10.55 (5.42)	10.35 (5.51)	10.06 (5.26)	0.11
**Mean maternal stress (PSS-10) score during child's infancy**	10.32 (6.11)	10.19 (5.93)	9.96 (5.99)	9.89 (5.91)	0.75

**Mean maternal stress (PSS-10) score at time CBCL was completed**	--	11.34 (6.11)	11.78 (6.28)	13.14 (6.37)	--
**Mean maternal depression (EPDS[d]) score during pregnancy**	4.25 (3.32)	3.91 (3.06)	3.82 (3.11)	3.71 (3.13)	0.18
**Mean maternal depression (EPDS) score during child's infancy**	3.85 (3.47)	3.81 (3.28)	3.57 (3.29)	3.61 (3.22)	0.76
**Mean maternal depression (EPDS) score at time CBCL was completed**	--	4.26 (3.48)	4.64 (3.86)	5.10 (4.09)	--

Supplemental Table 4. Categorical child demographics for all IKIDS participants with exposure data available and an infant enrolled at birth and each subsample who provided CBCL data when children were 2, 3, and 4 years.

**Child Demographics**	**Participants with exposure data** *(n = 514)*	**Participants with exposure and CBCL data at 2 yrs** *(n = 285)*	**Participants with exposure and CBCL data at 3 yrs** *(n = 240)*	**Participants with exposure and CBCL data at 4 yrs** *(n = 195)*	**p-value†**
	**N (%)**	**N (%)**	**N (%)**	**N (%)**	
**Child sex**					0.94
Male	252 (49.0)	136 (47.7)	126 (49.4)	98 (50.3)	
Female	262 (51.0)	149 (52.3)	129 (50.6)	97 (49.7)	
**Child race & ethnicity**					0.45
White, Non-Hispanic	387 (75.3)	229 (80.4)	207 (81.2)	157 (80.5)	
Other	125 (24.3)	56 (19.6)	48 (18.8)	38 (19.5)	
Unknown/Missing	2 (0.4)	0 (0.0)	0 (0.0)	0 (0.0)	
**Delivery type**					0.81
Vaginal	138 (26.8)	206 (72.3)	183 (71.8)	136 (69.7)	
Cesarean section	357 (69.5)	70 (24.6)	63 (24.7)	51 (26.2)	
Unknown/Missing	19 (3.7)	9 (3.1)	9 (3.5)	8 (4.1)	

†p-values for categorical variables estimated using Chi-square tests					
Wilcoxon-sum rank tests for continuous variables (no differences in pairwise comparisons)					

Supplemental Figure 2. Bayesian Kernel Machine Regression results for the Total Problems Scores (top row), Internalizing Problems Scores (middle row), and Externalizing Problems Scores (bottom row) at ages 2 (left column), 3 (middle column), and 4 (right column). The parabens mixture was not associated with the Total Problems score at age 2 (A), 3 (B), or 4 (C). It was also not associated with Internalizing Problems scores at 2 (D) or 3 years of age (E), but there was a positive association at age 4 (F). The mixture was also positively associated with Externalizing Problems scores at age 2 (G), but not at ages 3 (H) or 4 (I).

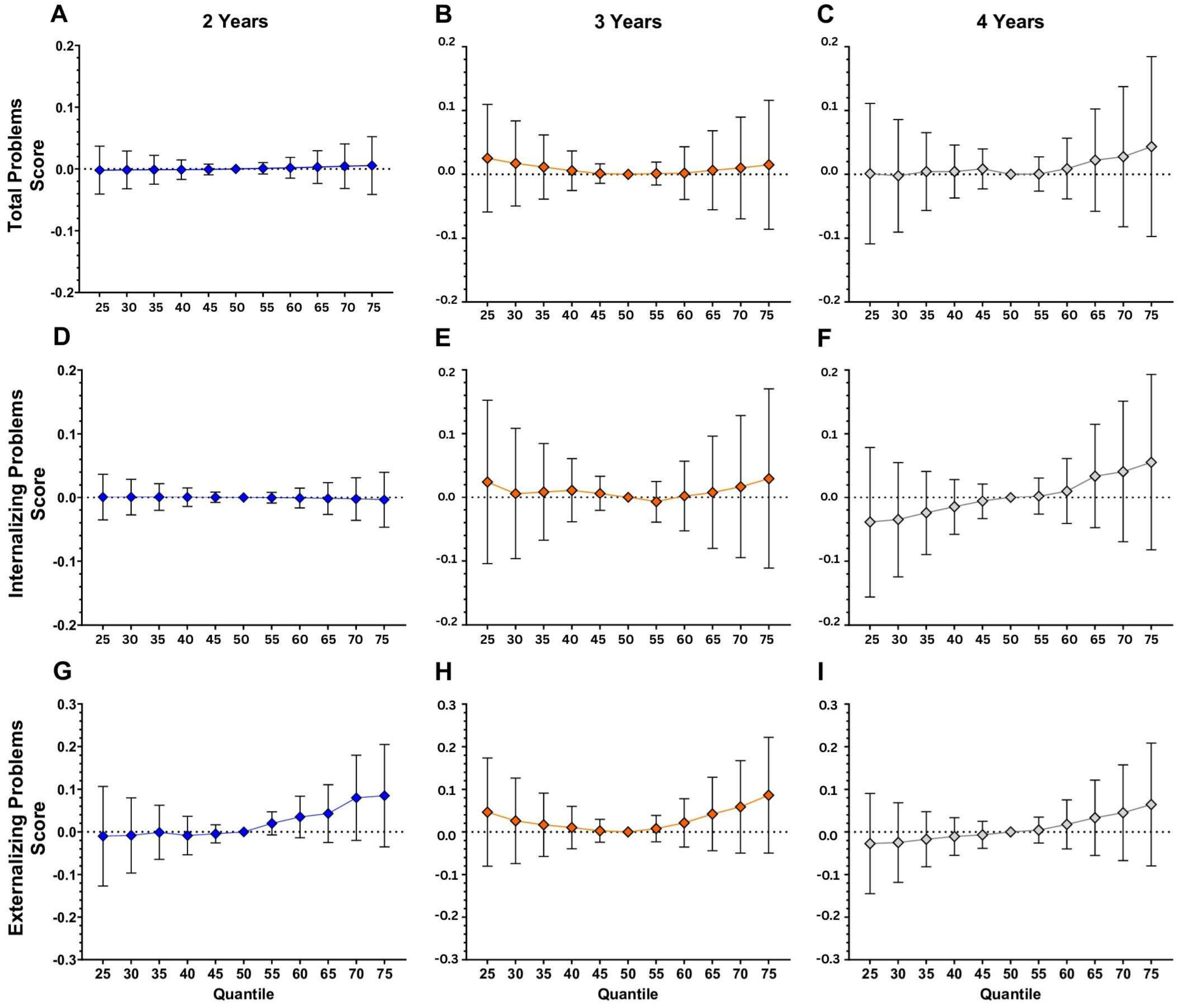

Supplemental Figure 3. Bayesian Kernel Machine Regression results for the Withdrawn Behavior (A), Attention Problems (B), and DSM ADHD Behaviors (C) subscales at age 2. The mixture was not associated with (A) Withdrawn Behavior or (B) Attention Problems scores, but it was positively associated with (C) DSM ADHD Behaviors scores.

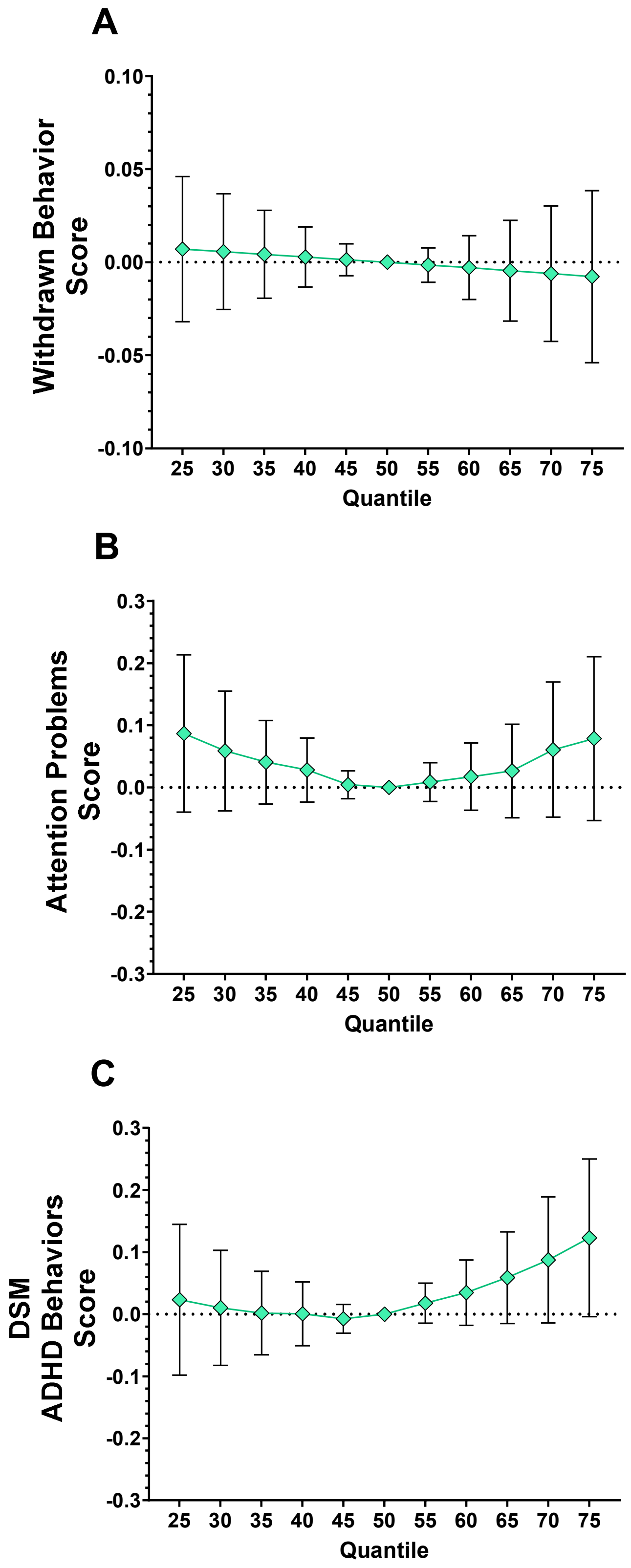
A
Withdrawn Behavior Score
0.10
0.05
0.00
-0.05
-0.10
25 30 35 40 45 50 55 60 65 70 75
Quantile
B
Attention Problems Score
0.3
0.2
0.1
0.0
-0.1
-0.2
-0.3
25 30 35 40 45 50 55 60 65 70 75
Quantile
C
DSM ADHD Behaviors Score
0.3
0.2
0.1
0.0
-0.1
-0.2
-0.3
25 30 35 40 45 50 55 60 65 70 75
Quantile

Supplemental Figure 4. Bayesian Kernel Machine Regression results for the Aggressive Problems subscale at age 3. There was no clear association of the mixture with Aggressive Problems scores.

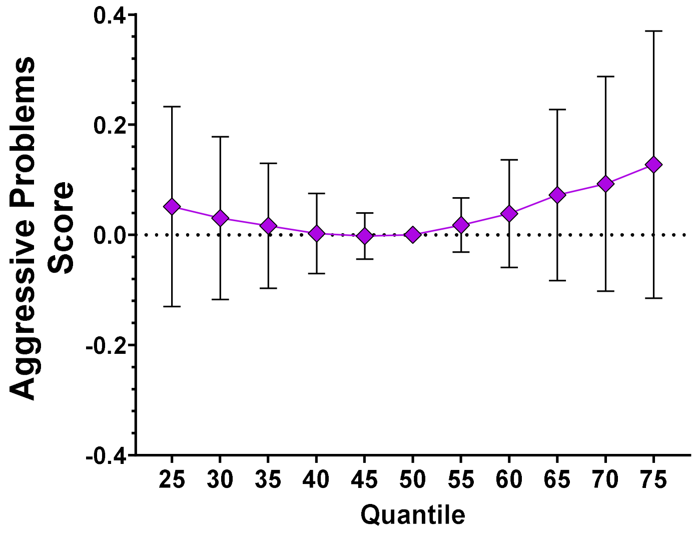

Supplemental Figure 5. Bayesian Kernel Machine Regression results for the Anxious/Depressed Problems (A), Somatic Complaints (B), Withdrawn Behavior (C), Pervasive Developmental Problems (D), and DSM Anxiety Problems (E) subscale scores at age 4. Only Anxious/Depressed Problems were associated with the mixture at 4 years of age.

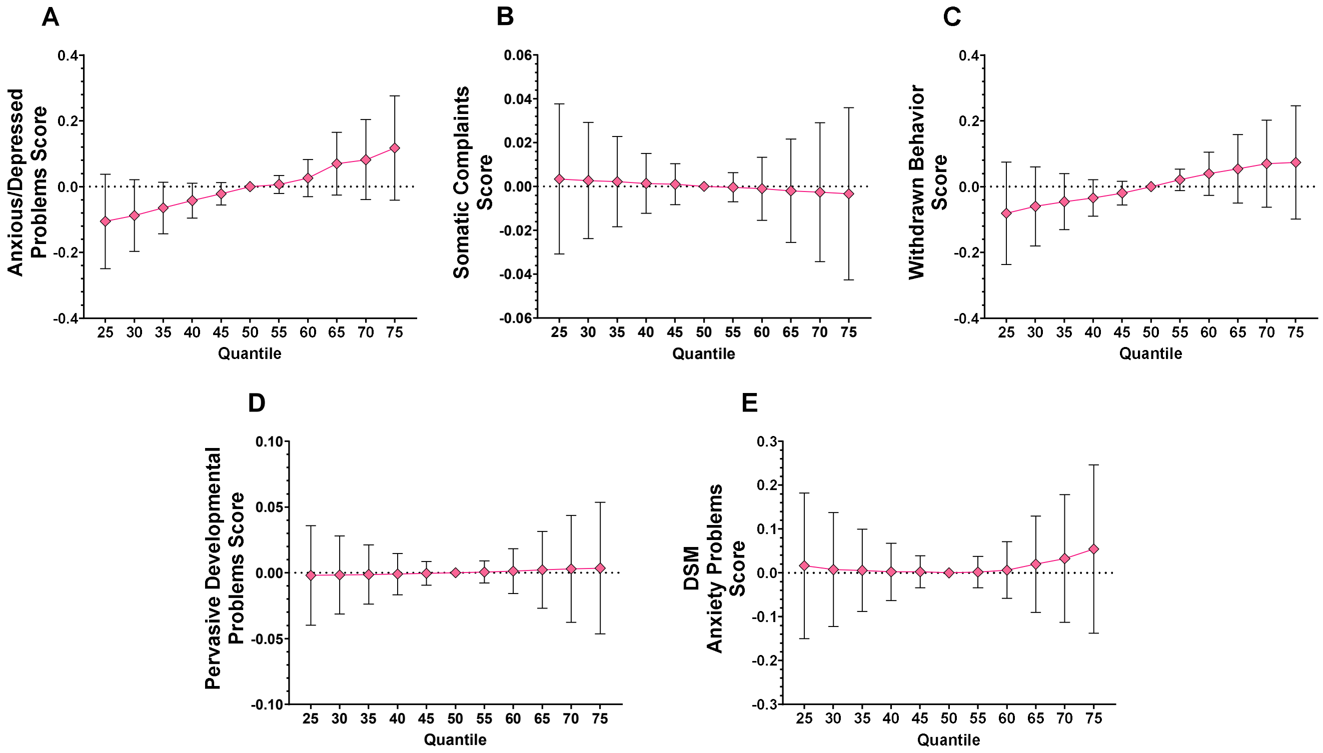

Supplemental Table 5. Sensitivity analysis: Multivariable linear regression analyses of the relation (β estimate and 95% confidence interval) of gestational paraben exposure with CBCL scores at ages 2, 3, and 4 years with any maternal alcohol use during pregnancy included in models. +p<0.1, *p<0.05, **p<0.01

		**CBCL completed at 2 years**	**CBCL completed at 3 years**	**CBCL completed at 4 years**
**Exposure**	**Outcome**	*n = 285*	*n = 255*	*n = 195*
Ethylparaben	Total Problems Score	0.779 (-0.154, 1.711)	0.623 (-0.338, 1.584)	0.295 (-0.991, 1.580)
	Internalizing Problems	-0.058 (-0.309, 0.193)	0.005 (-0.272, 0.281)	0.042 (-0.356, 0.440)
	Emotionally Reactive	0.030 (-0.069, 0.130)	0.060 (-0.056, 0.175)	0.011 (-0.145, 0.166)
	Anxious/Depressed	-0.032 (-0.132, 0.068)	0.038 (-0.066, 0.141)	0.028 (-0.117, 0.173)
	Somatic Complaints	-0.044 (-0.133, 0.045)	-0.072 (-0.169, 0.025)	0.020 (-0.106, 0.145)
	Withdrawn	-0.025 (-0.100, 0.050)	-0.039 (-0.111, 0.033)	0.041 (-0.059, 0.142)
	Pervasive Developmental Problems	-0.045 (-0.176, 0.087)	-0.043 (-0.169, 0.083)	0.031 (-0.143, 0.205)
	Anxiety Problems	-0.033 (-0.156, 0.089)	0.056 (-0.066, 0.178)	0.061 (-0.112, 0.235)
	Affective Problems	0.013 (-0.089, 0.114)	0.062 (-0.035, 0.159)	0.053 (-0.089, 0.195)
	Sleep Problems	0.023 (-0.123, 0.169)	0.085 (-0.066, 0.236)	0.017 (-0.184, 0.218)
	Externalizing Problems	**0.415 (-0.009, 0.838)+**	**0.416 (-0.012, 0.845)+**	0.174 (-0.350, 0.699)
	Attention Problems	**0.098 (-0.015, 0.211)+**	0.035 (-0.066, 0.136)	0.003 (-0.118, 0.124)
	Aggressive Behavior	**0.299 (-0.049, 0.647)+**	**0.375 (0.010, 0.739)***	0.162 (-0.294, 0.618)
	ADHD Problems	**0.213 (0.056, 0.371)****	0.033 (-0.120, 0.187)	-0.013 (-0.197, 0.172)
	Oppositional Defiant Problems	0.112 (-0.037, 0.262)	**0.248 (0.090, 0.406)****	0.103 (-0.085, 0.291)
Methylparaben	Total Problems Score	0.277 (-0.901, 1.454)	-0.119 (-1.345, 1.107)	0.172 (-1.446, 1.790)
	Internalizing Problems	-0.034 (-0.346, 0.278)	0.043 (-0.315, 0.401)	0.245 (-0.259, 0.750)
	Emotionally Reactive	0.068 (-0.057, 0.192)	0.004 (-0.144, 0.153)	0.136 (-0.06, 0.332)
	Anxious/Depressed	0.040 (-0.083, 0.162)	0.086 (-0.051, 0.222)	**0.200 (0.017, 0.382)***
	Somatic Complaints	-0.049 (-0.159, 0.062)	0.023 (-0.106, 0.151)	-0.077 (-0.240, 0.086)
	Withdrawn	**-0.097 (-0.190, -0.005)***	-0.038 (-0.132, 0.057)	0.046 (-0.081, 0.173)
	Pervasive Developmental Problems	-0.097 (-0.259, 0.065)	-0.013 (-0.178, 0.151)	0.033 (-0.187, 0.253)
	Anxiety Problems	-0.010 (-0.161, 0.142)	-0.029 (-0.189, 0.131)	0.085 (-0.134, 0.304)
	Affective Problems	-0.059 (-0.185, 0.067)	-0.010 (-0.137, 0.117)	0.060 (-0.123, 0.243)
	Sleep Problems	-0.084 (-0.265, 0.097)	-0.114 (-0.309, 0.081)	-0.129 (-0.382, 0.124)
	Externalizing Problems	0.194 (-0.341, 0.729)	0.059 (-0.505, 0.622)	0.327 (-0.339, 0.993)
	Attention Problems	-0.011 (-0.152, 0.129)	-0.024 (-0.156, 0.109)	-0.122 (-0.275, 0.031)
	Aggressive Behavior	0.163 (-0.275, 0.600)	0.085 (-0.394, 0.563)	0.403 (-0.177, 0.983)
	ADHD Problems	0.031 (-0.165, 0.227)	-0.034 (-0.235, 0.166)	-0.046 (-0.283, 0.191)
	Oppositional Defiant Problems	0.072 (-0.115, 0.259)	0.089 (-0.121, 0.299)	0.148 (-0.093, 0.389)
Propylparaben	Total Problems Score	-0.355 (-1.290, 0.580)	-0.479 (-1.457, 0.499)	-0.307 (-1.634, 1.020)
	Internalizing Problems	-0.131 (-0.377, 0.115)	-0.090 (-0.375, 0.195)	0.095 (-0.318, 0.508)
	Emotionally Reactive	0.018 (-0.081, 0.117)	-0.029 (-0.147, 0.089)	0.048 (-0.110, 0.205)
	Anxious/Depressed	-0.036 (-0.133, 0.062)	-0.010 (-0.117, 0.098)	0.121 (-0.026, 0.268)
	Somatic Complaints	-0.067 (-0.155, 0.021)	0.021 (-0.080, 0.123)	-0.051 (-0.184, 0.081)
	Withdrawn	-0.061 (-0.135, 0.013)	-0.053 (-0.128, 0.021)	0.049 (-0.052, 0.151)
	Pervasive Developmental Problems	-0.029 (-0.159, 0.102)	-0.032 (-0.163, 0.098)	0.022 (-0.156, 0.200)
	Anxiety Problems	-0.081 (-0.200, 0.039)	-0.081 (-0.207, 0.045)	0.041 (-0.135, 0.217)
	Affective Problems	-0.069 (-0.169, 0.032)	-0.044 (-0.144, 0.057)	0.021 (-0.124, 0.166)
	Sleep Problems	-0.108 (-0.253, 0.037)	-0.072 (-0.226, 0.081)	-0.060 (-0.260, 0.140)
	Externalizing Problems	-0.098 (-0.527, 0.331)	-0.079 (-0.529, 0.371)	0.184 (-0.357, 0.724)
	Attention Problems	-0.069 (-0.181, 0.043)	-0.048 (-0.152, 0.057)	-0.079 (-0.202, 0.045)
	Aggressive Behavior	-0.048 (-0.399, 0.303)	-0.032 (-0.413, 0.350)	0.188 (-0.280, 0.656)
	ADHD Problems	-0.020 (-0.176, 0.137)	-0.053 (-0.212, 0.106)	-0.028 (-0.217, 0.162)
	Oppositional Defiant Problems	-0.030 (-0.181, 0.120)	0.039 (-0.127, 0.205)	0.026 (-0.168, 0.220)

Supplemental Table 6. Sensitivity analysis: Multivariable linear regression analyses of the relation (β estimate and 95% confidence interval) of gestational paraben exposure with CBCL scores at ages 2, 3, and 4 years with mothers who smoked during pregnancy excluded. +p<0.1, *p<0.05, **p<0.01

		**CBCL completed at**	**CBCL completed at**	**CBCL completed at**
		**2 years**	**3 years**	**4 years**
**Exposure**	**Outcome**	*n = 285*	*n = 255*	*n = 195*
Ethylparaben	Total Problems Score	0.804 (-0.145, 1.753)+	0.477 (-0.529, 1.483)	0.244 (-1.072, 1.56)
	Internalizing Problems	-0.056 (-0.311, 0.199)	0.009 (-0.275, 0.293)	0.024 (-0.381, 0.429)
	Emotionally Reactive	0.041 (-0.06, 0.142)	0.066 (-0.055, 0.187)	0.006 (-0.153, 0.165)
	Anxious/Depressed	-0.033 (-0.135, 0.069)	0.027 (-0.078, 0.132)	0.017 (-0.132, 0.164)
	Somatic Complaints	-0.053 (-0.143, 0.037)	-0.059 (-0.16, 0.042)	0.02 (-0.107, 0.147)
	Withdrawn	-0.023 (-0.099, 0.052)	-0.044 (-0.119, 0.032)	0.038 (-0.065, 0.14)
	Pervasive Developmental Problems	-0.035 (-0.169, 0.098)	-0.043 (-0.174, 0.087)	0.009 (-0.169, 0.186)
	Anxiety Problems	-0.032 (-0.156, 0.093)	0.06 (-0.066, 0.186)	0.053 (-0.123, 0.229)
	Affective Problems	0.011 (-0.093, 0.115)	0.068 (-0.033, 0.169)	0.064 (-0.082, 0.21)
	Sleep Problems	0.007 (-0.142, 0.155)	0.086 (-0.07, 0.243)	0.035 (-0.169, 0.24)
	Externalizing Problems	**0.411 (-0.021, 0.844)+**	0.323 (-0.125, 0.77)	0.122 (-0.412, 0.657)
	Attention Problems	**0.097 (-0.018, 0.212)+**	0.005 (-0.101, 0.11)	-0.006 (-0.127, 0.115)
	Aggressive Behavior	0.295 (-0.06, 0.65)	0.31 (-0.07, 0.69)	0.124 (-0.339, 0.587)
	ADHD Problems	**0.206 (0.045, 0.367)***	-0.014 (-0.173, 0.145)	-0.034 (-0.22, 0.153)
	Oppositional Defiant Problems	0.112 (-0.041, 0.265)	**0.223 (0.058, 0.388)****	0.102 (-0.09, 0.294)
Methylparaben	Total Problems Score	0.281 (-0.903, 1.466)	-0.04 (-1.279, 1.198)	0.378 (-1.281, 2.037)
	Internalizing Problems	-0.036 (-0.349, 0.277)	0.058 (-0.297, 0.413)	0.281 (-0.233, 0.796)
	Emotionally Reactive	0.069 (-0.056, 0.195)	0.014 (-0.136, 0.165)	0.149 (-0.052, 0.349)
	Anxious/Depressed	0.038 (-0.085, 0.162)	0.073 (-0.06, 0.207)	**0.209 (0.023, 0.395)***
	Somatic Complaints	-0.053 (-0.164, 0.058)	0.038 (-0.091, 0.166)	-0.062 (-0.227, 0.104)
	Withdrawn	**-0.095 (-0.188, -0.003)***	-0.036 (-0.132, 0.06)	0.044 (-0.086, 0.174)
	Pervasive Developmental Problems	-0.094 (-0.257, 0.069)	-0.005 (-0.17, 0.159)	0.029 (-0.196, 0.254)
	Anxiety Problems	-0.016 (-0.168, 0.137)	-0.021 (-0.181, 0.139)	0.111 (-0.112, 0.333)
	Affective Problems	-0.053 (-0.18, 0.074)	-0.01 (-0.139, 0.119)	0.074 (-0.114, 0.261)
	Sleep Problems	-0.077 (-0.259, 0.105)	-0.096 (-0.292, 0.101)	-0.112 (-0.37, 0.145)
	Externalizing Problems	0.204 (-0.335, 0.744)	0.092 (-0.477, 0.66)	0.406 (-0.273, 1.085)
	Attention Problems	-0.012 (-0.154, 0.13)	-0.028 (-0.162, 0.106)	-0.124 (-0.277, 0.03)
	Aggressive Behavior	0.171 (-0.271, 0.613)	0.12 (-0.363, 0.603)	0.48 (-0.109, 1.069)
	ADHD Problems	0.031 (-0.167, 0.229)	-0.03 (-0.232, 0.171)	-0.039 (-0.279, 0.2)
	Oppositional Defiant Problems	0.08 (-0.11, 0.269)	0.102 (-0.11, 0.313)	0.167 (-0.079, 0.413)
Propylparaben	Total Problems Score	-0.339 (-1.284, 0.606)	-0.396 (-1.39, 0.598)	-0.094 (-1.446, 1.259)
	Internalizing Problems	-0.131 (-0.379, 0.118)	-0.105 (-0.389, 0.179)	0.134 (-0.285, 0.553)
	Emotionally Reactive	0.015 (-0.085, 0.115)	-0.028 (-0.148, 0.092)	0.059 (-0.101, 0.22)
	Anxious/Depressed	-0.035 (-0.133, 0.064)	-0.03 (-0.136, 0.076)	0.131 (-0.018, 0.28)
	Somatic Complaints	-0.063 (-0.152, 0.025)	0.03 (-0.073, 0.132)	-0.042 (-0.176, 0.092)
	Withdrawn	-0.062 (-0.136, 0.013)	-0.056 (-0.132, 0.02)	0.049 (-0.054, 0.152)
	Pervasive Developmental Problems	-0.032 (-0.164, 0.099)	-0.035 (-0.166, 0.096)	0.027 (-0.154, 0.208)
	Anxiety Problems	-0.082 (-0.203, 0.039)	-0.08 (-0.206, 0.047)	0.061 (-0.117, 0.239)
	Affective Problems	-0.067 (-0.169, 0.035)	-0.049 (-0.151, 0.053)	0.026 (-0.122, 0.174)
	Sleep Problems	-0.095 (-0.241, 0.052)	-0.056 (-0.211, 0.099)	-0.057 (-0.259, 0.146)

	Externalizing Problems	-0.086 (-0.521, 0.349)	-0.017 (-0.473, 0.44)	0.256 (-0.291, 0.803)
	Attention Problems	-0.069 (-0.183, 0.044)	-0.042 (-0.148, 0.064)	-0.07 (-0.193, 0.053)
	Aggressive Behavior	-0.038 (-0.394, 0.319)	0.024 (-0.364, 0.411)	0.247 (-0.225, 0.72)
	ADHD Problems	-0.013 (-0.171, 0.145)	-0.035 (-0.195, 0.126)	-0.012 (-0.202, 0.178)
	Oppositional Defiant Problems	-0.024 (-0.177, 0.128)	0.062 (-0.107, 0.23)	0.04 (-0.157, 0.237)

Supplemental Table 7. Sensitivity analysis: Multivariable linear regression analyses of the relation (β estimate and 95% confidence interval) of gestational paraben exposure with CBCL scores at ages 2, 3, and 4 years with influential cases (identified with a Cook's distance >0.06) excluded. +p<0.1, *p<0.05, **p<0.01

		**CBCL completed at**	**CBCL completed at**	**CBCL completed at**
		**2 years**	**3 years**	**4 years**
**Exposure**	**Outcome**	*n = 285*	*n = 255*	*n = 195*
Ethylparaben	Total Problems Score	0.667 (-0.615; 1.949)	0.518 (-0.725; 1.760)	0.011 (-1.391; 1.413)
	Internalizing Problems	-0.093 (-0.446; 0.259)	-0.072 (-0.449; 0.305)	-0.152 (-0.578; 0.274)
	Emotionally Reactive	0.005 (-0.134; 0.145)	0.039 (-0.124; 0.203)	-0.010 (-0.187; 0.167)
	Anxious/Depressed	-0.004 (-0.153; 0.146)	0.047 (-0.099; 0.192)	-0.019 (-0.178; 0.139)
	Somatic Complaints	-0.062 (-0.185; 0.061)	-0.099 (-0.233; 0.035)	-0.020 (-0.163; 0.123)
	Withdrawn	-0.032 (-0.115; 0.051)	-0.071 (-0.178; 0.036)	-0.023 (-0.102; 0.057)
	Pervasive Developmental Problems	-0.063 (-0.224; 0.098)	-0.132 (-0.303; 0.038)	-0.022 (-0.217; 0.173)
	Anxiety Problems	0.055 (-0.127; 0.236)	0.067 (-0.088; 0.222)	0.051 (-0.138; 0.240)
	Affective Problems	0.032 (-0.096; 0.160)	0.018 (-0.109; 0.145)	0.007 (-0.149; 0.162)
	Sleep Problems	0.095 (-0.088; 0.260)	0.047 (-0.151; 0.244)	0.035 (-0.186; 0.256)
	Externalizing Problems	0.357 (-0.220; 0.933)	**0.481 (-0.080; 1.041)+**	0.176 (-0.399; 0.751)
	Attention Problems	0.056 (-0.100; 0.211)	0.052 (-0.081; 0.184)	-0.008 (-0.135; 0.120)
	Aggressive Behavior	0.312 (-0.158; 0.782)	**0.420 (-0.057; 0.898)+**	0.156 (-0.348; 0.660)
	ADHD Problems	**0.176 (-0.030; 0.381)+**	0.083 (-0.122; 0.287)	-0.042 (-0.247; 0.163)
	Oppositional Defiant Problems	0.133 (-0.080; 0.345)	**0.300 (-0.086; 0.514)****	0.134 (-0.070; 0.338)
Methylparaben	Total Problems Score	0.516 (-0.212; 0.243)	-0.275 (-1.882; 1.331)	-0.133 (-2.009; 1.743)
	Internalizing Problems	0.050 (-0.417; 0.517)	-0.002 (-0.501; 0.498)	0.153 (-0.422; 0.728)
	Emotionally Reactive	0.023 (-0.167; 0.213)	-0.040 (-0.257; 0.177)	0.111 (-0.127; 0.349)
	Anxious/Depressed	0.104 (-0.093; 0.301)	**0.166 (-0.025; 0.358)+**	**0.185 (-0.028; 0.328)+**
	Somatic Complaints	-0.077 (-0.245; 0.091)	-0.071 (-0.250; 0.108)	-0.110 (-0.306; 0.086)
	Withdrawn	-0.016 (-0.130; 0.097)	-0.043 (-0.186; 0.099)	0.064 (-0.042; 0.171)
	Pervasive Developmental Problems	-0.076 (-0.294; 0.142)	-0.052 (-0.280; 0.177)	0.019 (-0.244; 0.282)
	Anxiety Problems	0.114 (-0.124; 0.353)	-0.007 (-0.213; 0.199)	0.102 (-0.154; 0.359)
	Affective Problems	0.014 (-0.160; 0.188)	0.015 (-0.154; 0.184)	0.067 (-0.145; 0.280)
	Sleep Problems	0.077 (-0.172; 0.325)	-0.191 (-0.446; 0.064)	-0.097 (-0.392; 0.199)
	Externalizing Problems	0.096 (-0.704; 0.896)	0.159 (-0.590; 0.907)	0.240 (-0.546; 1.025)
	Attention Problems	-0.062 (-0.273; 0.150)	0.012 (-0.165; 0.189)	**-0.169 (-0.340; 0.003)+**
	Aggressive Behavior	0.189 (-0.464; 0.842)	0.170 (-0.466; 0.806)	0.317 (-0.373; 1.006)
	ADHD Problems	-0.015 (-0.295; 0.264)	-0.045 (-0.318; 0.228)	-0.131 (-0.411; 0.148)
	Oppositional Defiant Problems	0.085 (-0.208; 0.377)	0.111 (-0.179; 0.401)	0.156 (-0.124; 0.436)
Propylparaben	Total Problems Score	-0.649 (-2.098; 0.801)	-0.970 (-2.288; 0.348)	-1.065 (-2.601; 0.471)
	Internalizing Problems	-0.204 (-0.584; 0.177)	-0.203 (-0.615; 0.209)	-0.110 (-0.584; 0.364)
	Emotionally Reactive	-0.010 (-0.167; 0.146)	-0.078 (-0.257; 0.101)	-0.024 (-0.218; 0.170)
	Anxious/Depressed	-0.057 (-0.218; 0.104)	0.035 (-0.125; 0.194)	0.073 (-0.101; 0.248)

	Somatic Complaints	-0.122 (-0.259; 0.014)+	-0.063 (-0.211; 0.084)	-0.093 (-0.253; 0.067)
	Withdrawn	-0.023 (-0.117; 0.070)	-0.087 (-0.204; 0.030)	0.027 (-0.059; 0.114)
	Pervasive Developmental Problems	-0.052 (-0.233; 0.130)	-0.102 (-0.291; 0.086)	-0.025 (-0.238; 0.188)
	Anxiety Problems	-0.080 (-0.276; 0.116)	-0.088 (-0.258; 0.082)	0.015 (-0.194; 0.223)
	Affective Problems	-0.036 (-0.179; 0.107)	-0.077 (-0.216; 0.062)	-0.043 (-0.215; 0.128)
	Sleep Problems	-0.005 (-0.211; 0.201)	-0.126 (-0.334; 0.083)	-0.068 (-0.303; 0.168)
	Externalizing Problems	-0.324 (-0.998; 0.350)	-0.182 (-0.806; 0.442)	-0.037 (-0.685; 0.610)
	Attention Problems	-0.109 (-0.284; 0.065)	-0.049 (-0.196; 0.097)	-0.156 (-0.295; 0.017)*
	Aggressive Behavior	-0.160 (-0.706; 0.387)	-0.104 (-0.633; 0.425)	-0.002 (-0.568; 0.563)
	ADHD Problems	-0.048 (-0.278; 0.182)	-0.080 (-0.306; 0.146)	-0.133 (-0.360; 0.093)
	Oppositional Defiant Problems	-0.114 (-0.357; 0.130)	0.002 (-0.238; 0.242)	-0.023 (-0.252; 0.205)

Supplemental Table 8. Multivariable linear regression analyses of the relation (β estimate and 95% confidence interval) of gestational paraben exposure with CBCL scores at 2 years of age, stratified. +p<0.1, *p<0.05, **p<0.01

				**CBCL completed at 2 years**	
Adjusted[1]				**Females**	**Males**
**Outcome**	**Exposure**	Main effect (n = 285)	Sex inter.	*n = 149*	*n = 136*
	Total Problems Score			0.912 (-0.403, 2.226)	0.649 (-0.626, 1.923)
	Internalizing Problems			0.072 (-0.280, 0.423)	-0.181 (-0.525, 0.163)
	Emotionally Reactive			0.057 (-0.085, 0.199)	0.003 (-0.132, 0.138)
	Anxious/Depressed			0.016 (-0.124, 0.157)	-0.078 (-0.215, 0.060)
	Somatic Complaints			-0.052 (-0.180, 0.076)	-0.031 (-0.152, 0.091)
	Withdrawn	-0.025 (-0.100, 0.050)	+	0.048 (-0.058, 0.154)	**-0.091 (-0.192, 0.011)+**
	Pervasive Dev. Probs.			0.020 (-0.167, 0.207)	-0.100 (-0.278, 0.078)
EtPB	Anxiety Problems			-0.050 (-0.223, 0.122)	-0.015 (-0.183, 0.153)
	Affective Problems			0.076 (-0.070, 0.221)	-0.049 (-0.187, 0.089)
	Sleep Problems			0.060 (-0.149, 0.268)	-0.017 (-0.216, 0.183)
	Externalizing Problems			0.402 (-0.207, 1.012)	0.407 (-0.168, 0.982)
	Attention Problems			0.022 (-0.140, 0.183)	**0.161 (0.008, 0.314)***
	Aggressive Behavior			0.364 (-0.135, 0.863)	0.228 (-0.245, 0.700)
	ADHD Problems			0.195 (-0.030, 0.420)	**0.226 (0.012, 0.440)***
	Oppos. Defiant Probs.			0.129 (-0.087, 0.344)	0.089 (-0.115, 0.293)
	Total Problems Score			-0.373 (-1.889, 1.144)	1.261 (-0.578, 3.100)
	Internalizing Problems			-0.019 (-0.426, 0.388)	-0.060 (-0.537, 0.418)
	Emotionally Reactive			0.019 (-0.144, 0.183)	0.136 (-0.054, 0.327)
	Anxious/Depressed			0.063 (-0.096, 0.222)	0.004 (-0.186, 0.195)
	Somatic Complaints			-0.036 (-0.181, 0.109)	-0.075 (-0.246, 0.097)
	Withdrawn	**-0.098 (-0.190, -0.006)***	#	-0.028 (-0.147, 0.092)	**-0.197 (-0.339, -0.055)****
	Pervasive Dev. Probs.			-0.051 (-0.261, 0.160)	-0.165 (-0.416, 0.086)
MePB	Anxiety Problems			-0.039 (-0.237, 0.159)	0.024 (-0.209, 0.256)
	Affective Problems			-0.031 (-0.196, 0.133)	-0.092 (-0.287, 0.103)
	Sleep Problems			-0.095 (-0.331, 0.141)	-0.060 (-0.340, 0.220)
	Externalizing Problems			-0.085 (-0.774, 0.604)	0.648 (-0.194, 1.491)
	Attention Problems			-0.112 (-0.292, 0.068)	0.152 (-0.070, 0.373)
	Aggressive Behavior			0.033 (-0.540, 0.606)	0.361 (-0.312, 1.035)
	ADHD Problems			-0.053 (-0.306, 0.199)	0.160 (-0.148, 0.468)
	Oppos. Defiant Probs.			-0.007 (-0.252, 0.239)	0.196 (-0.093, 0.486)

PrPB	Total Problems Score			-0.737 (-1.998, 0.525)	0.111 (-1.268, 1.490)
	Internalizing Problems			-0.141 (-0.477, 0.195)	-0.121 (-0.478, 0.236)
	Emotionally Reactive			-0.005 (-0.140, 0.131)	0.044 (-0.100, 0.187)
	Anxious/Depressed			-0.009 (-0.142, 0.124)	-0.066 (-0.209, 0.076)
	Somatic Complaints			-0.116 (-0.237, 0.004)	-0.013 (-0.142, 0.115)
	Withdrawn		#	0.003 (-0.098, 0.103)	**-0.134 (-0.241, -0.026)***
	Pervasive Dev. Probs.			0.010 (-0.168, 0.188)	-0.072 (-0.262, 0.118)
	Anxiety Problems			-0.079 (-0.243, 0.085)	-0.084 (-0.258, 0.090)
	Affective Problems			-0.056 (-0.193, 0.082)	-0.082 (-0.228, 0.065)
	Sleep Problems			-0.124 (-0.321, 0.073)	-0.087 (-0.299, 0.124)
	Externalizing Problems			-0.255 (-0.836, 0.327)	0.103 (-0.533, 0.739)
	Attention Problems			-0.130 (-0.281, 0.022)	0.006 (-0.160, 0.172)
	Aggressive Behavior			-0.112 (-0.594, 0.370)	0.031 (-0.480, 0.542)
	ADHD Problems			-0.086 (-0.297, 0.124)	0.061 (-0.169, 0.292)
	Oppos. Defiant Probs.			-0.103 (-0.308, 0.102)	0.058 (-0.162, 0.277)

Supplemental Table 9. Multivariable linear regression analyses of the relation (β estimate and 95% confidence interval) of gestational paraben exposure with CBCL scores at 3 years of age, stratified by sex. +p<0.1, *p<0.05, **p<0.01

				CBCL completed at 3 years	
				**Females**	**Males**
**Outcome**	**Exposure**	Main effect N=255		*n = 129*	*n = 126*
EtPB	Total Problems Score			0.718 (-0.803, 2.240)	0.566 (-0.654, 1.787)
	Internalizing Problems			0.064 (-0.356, 0.483)	-0.037 (-0.399, 0.325)
	Emotionally Reactive			0.032 (-0.142, 0.207)	0.081 (-0.070, 0.232)
	Anxious/Depressed			0.097 (-0.060, 0.255)	-0.010 (-0.147, 0.126)
	Somatic Complaints			-0.044 (-0.190, 0.103)	-0.092 (-0.221, 0.036)
	Withdrawn			-0.027 (-0.136, 0.082)	0.046 (-0.141, 0.050)
	Pervasive Dev. Probs.			-0.031 (-0.224, 0.161)	-0.052 (-0.217, 0.113)
	Anxiety Problems			0.024 (-0.160, 0.209)	0.078 (-0.083, 0.239)
	Affective Problems			0.106 (-0.040, 0.252)	0.029 (-0.098, 0.157)
	Sleep Problems			0.020 (-0.213, 0.253)	0.131 (-0.064, 0.326)
	Externalizing Problems			0.342 (-0.316, 0.999)	0.479 (-0.081, 1.039)
	Attention Problems			-0.003 (-0.156, 0.150)	0.070 (-0.063, 0.204)
	Aggressive Behavior			0.340 (-0.217, 0.896)	0.405 (-0.072, 0.881)
	ADHD Problems			0.034 (-0.199, 0.267)	0.039 (-0.164, 0.242)
	Oppos. Defiant Probs.			0.231 (-0.009, 0.471)	**0.266 (0.058, 0.474)***
MePB	Total Problems Score			-0.430 (-2.091, 1.230)	0.292 (-1.531, 2.115)
	Internalizing Problems			-0.036 (-0.515, 0.442)	0.149 (-0.390, 0.688)
	Emotionally Reactive			-0.060 (-0.259, 0.139)	0.088 (-0.136, 0.312)
	Anxious/Depressed			0.110 (-0.072, 0.291)	0.047 (-0.158, 0.253)
	Somatic Complaints			0.030 (-0.140, 0.200)	0.015 (-0.181, 0.210)
	Withdrawn			-0.063 (-0.189, 0.063)	0.002 (-0.142, 0.146)
	Pervasive Dev. Probs.			-0.145 (-0.363, 0.074)	0.155 (-0.092, 0.402)
	Anxiety Problems			-0.086 (-0.299, 0.127)	0.041 (-0.202, 0.283)
	Affective Problems			-0.005 (-0.174, 0.164)	-0.015 (-0.208, 0.178)
	Sleep Problems			-0.199 (-0.460, 0.063)	-0.005 (-0.298, 0.289)

	Externalizing Problems			-0.122 (-0.876, 0.633)	0.323 (-0.526, 1.172)
	Attention Problems			-0.093 (-0.269, 0.083)	0.082 (-0.119, 0.283)
	Aggressive Behavior			-0.034 (-0.675, 0.607)	0.254 (-0.467, 0.976)
	ADHD Problems			-0.061 (-0.329, 0.208)	0.019 (-0.286, 0.323)
	Oppos. Defiant Probs.			-0.003 (-0.282, 0.276)	0.221 (-0.097, 0.539)
PrPB	Total Problems Score			-0.305 (-1.689, 1.079)	-0.641 (-2.010, 0.727)
	Internalizing Problems			-0.101 (-0.499, 0.297)	-0.077 (-0.480, 0.327)
	Emotionally Reactive			-0.043 (-0.210, 0.123)	-0.014 (-0.181, 0.152)
	Anxious/Depressed			0.006 (-0.144, 0.157)	-0.029 (-0.182, 0.125)
	Somatic Complaints			0.011 (-0.130, 0.152)	0.033 (-0.113, 0.179)
	Withdrawn			-0.045 (-0.149, 0.060)	-0.060 (-0.166, 0.047)
	Pervasive Dev. Probs.			-0.096 (-0.278, 0.086)	0.033 (-0.151, 0.218)
	Anxiety Problems			-0.118 (-0.294, 0.059)	-0.044 (-0.224, 0.136)
	Affective Problems			-0.027 (-0.167, 0.113)	-0.060 (-0.203, 0.083)
	Sleep Problems			-0.153 (-0.367, 0.061)	0.012 (-0.206, 0.231)
	Externalizing Problems			-0.038 (-0.674, 0.599)	0.103 (-0.533, 0.739)
	Attention Problems			-0.085 (-0.231, 0.060)	0.006 (-0.160, 0.172)
	Aggressive Behavior			0.032 (-0.508, 0.572)	0.031 (-0.480, 0.542)
	ADHD Problems			-0.009 (-0.234, 0.215)	0.061 (-0.169, 0.292)
	Oppos. Defiant Probs.			0.010 (-0.223, 0.242)	0.058 (-0.162, 0.277)

Supplemental Table 10. Multivariable linear regression analyses of the relation (β estimate and 95% confidence interval) of gestational paraben exposure with CBCL scores at 4 years of age, stratified by sex. +p<0.1, *p<0.05, **p<0.01

				**CBCL completed at 4 years**	
Adjusted[1]				**Females**	**Males**
**Outcome**	**Exposure**	Main effect N=195	Sex inter.	*n = 97*	*n = 98*
EtPB	Total Problems Score			1.469 (-0.724, 3.663)	-0.142 (-1.681, 1.396)
	Internalizing Problems			0.029 (-0.617, 0.676)	0.081 (-0.410, 0.572)
	Emotionally Reactive			0.051 (-0.197, 0.300)	-0.001 (-0.195, 0.194)
	Anxious/Depressed			-0.002 (-0.232, 0.227)	0.050 (-0.132, 0.231)
	Somatic Complaints			0.032 (-0.167, 0.230)	0.011 (-0.147, 0.169)
	Withdrawn			0.082 (-0.079, 0.242)	0.018 (-0.106, 0.143)
	Pervasive Dev. Probs.			0.182 (-0.092, 0.457)	-0.056 (-0.271, 0.159)
	Anxiety Problems			0.012 (-0.257, 0.282)	0.097 (-0.123, 0.317)
	Affective Problems			0.108 (-0.118, 0.334)	0.028 (-0.150, 0.206)
	Sleep Problems			0.021 (-0.309, 0.351)	0.020 (-0.226, 0.267)
	Externalizing Problems			0.536 (-0.304, 1.376)	-0.029 (-0.678, 0.620)
	Attention Problems			-0.003 (-0.197, 0.191)	0.013 (-0.138, 0.164)
	Aggressive Behavior			0.493 (-0.226, 1.212)	-0.036 (-0.605, 0.533)
	ADHD Problems			-0.018 (-0.310, 0.274)	-0.001 (-0.234, 0.232)
	Oppos. Defiant Probs.			0.196 (-0.101, 0.494)	0.056 (-0.180, 0.292)
MePB	Total Problems Score			-0.450 (-2.667, 1.766)	0.881 (-1.408, 3.170)
	Internalizing Problems			-0.052 (-0.736, 0.633)	0.582 (-0.139, 1.303)
	Emotionally Reactive			0.124 (-0.141, 0.388)	0.147 (-0.139, 0.433)
	Anxious/Depressed			0.066 (-0.178, 0.311)	**0.353 (0.090, 0.615)****
	Somatic Complaints			-0.181 (-0.402, 0.040)	0.037 (-0.194, 0.268)

	Withdrawn			-0.012 (-0.183, 0.159)	0.113 (-0.071, 0.296)
	Pervasive Dev. Probs.			-0.025 (-0.321, 0.271)	0.100 (-0.218, 0.419)
	Anxiety Problems		#	-0.106 (-0.398, 0.185)	0.306 (-0.008, 0.621)+
	Affective Problems			-0.045 (-0.291, 0.202)	0.176 (-0.086, 0.438)
	Sleep Problems			-0.238 (-0.580, 0.103)	-0.010 (-0.372, 0.353)
	Externalizing Problems			0.139 (-0.759, 1.038)	0.538 (-0.420, 1.496)
	Attention Problems			-0.086 (-0.293, 0.122)	-0.168 (-0.389, 0.054)
	Aggressive Behavior			0.131 (-0.648, 0.909)	0.717 (-0.119, 1.554)
	ADHD Problems			-0.064 (-0.383, 0.255)	-0.030 (-0.374, 0.314)
	Oppos. Defiant Probs.			0.025 (-0.300, 0.350)	0.282 (-0.065, 0.630)
PrPB	Total Problems Score			-0.434 (-2.354, 1.486)	-0.123 (-1.930, 1.683)
	Internalizing Problems			-0.068 (-0.657, 0.521)	0.257 (-0.313, 0.827)
	Emotionally Reactive			0.072 (-0.149, 0.294)	0.021 (-0.202, 0.244)
	Anxious/Depressed			0.044 (-0.160, 0.248)	0.201 (-0.007, 0.408)
	Somatic Complaints		#	**-0.167 (-0.354, 0.020)+**	0.058 (-0.124, 0.239)
	Withdrawn			0.049 (-0.093, 0.190)	0.049 (-0.094, 0.193)
	Pervasive Dev. Probs.			-0.012 (-0.263, 0.239)	0.056 (-0.192, 0.304)
	Anxiety Problems		#	-0.117 (-0.359, 0.124)	0.207 (-0.041, 0.455)
	Affective Problems			-0.044 (-0.248, 0.159)	0.086 (-0.118, 0.291)
	Sleep Problems			-0.188 (-0.466, 0.090)	0.070 (-0.212, 0.352)
	Externalizing Problems			0.231 (-0.529, 0.991)	0.137 (-0.615, 0.889)
	Attention Problems			-0.027 (-0.201, 0.147)	-0.132 (-0.304, 0.041)
	Aggressive Behavior			0.105 (-0.547, 0.757)	0.273 (-0.386, 0.932)
	ADHD Problems			-0.017 (-0.281, 0.248)	-0.041 (-0.310, 0.227)
	Oppos. Defiant Probs.			-0.037 (-0.309, 0.235)	0.088 (-0.185, 0.360)
